# An immune-resistance dual-axis framework prioritizes MAL2 for functional investigation in lung cancer

**DOI:** 10.3389/fphar.2026.1940144

**Published:** 2026-10-30

**Authors:** JingYi Zhang, Siqi Luo, Xinming Jing, Xianfeng Lu, Yanli Xiong

**Affiliations:** 1 Department of Oncology, Daping Hospital, Army Medical University, Chongqing, China; 2 Department of Hematology & Oncology, Jiangbei Campus, The First Affiliated Hospital of Army Medical University, Chongqing, China; 3 Chongqing Municipal Health Commission Key Laboratory of Intelligent Clinical Nutrition and Transformation, Chongqing, China

**Keywords:** immune checkpoint blockade, immunotherapy, integrative transcriptomics, lung cancer, MAL2, single-cell RNA sequencing, tumor microenvironment, tumor-intrinsic resistance

## Abstract

**Background:**

Immune checkpoint blockade has reshaped lung cancer treatment, yet durable benefit remains restricted to a subset of patients. Biomarkers incompletely capture interactions between immune activation and tumor-intrinsic resistance. Integrating immune and epithelial expression programs may help identify response-associated states and candidates for functional investigation.

**Methods:**

Public bulk transcriptomes were analyzed with format-specific preprocessing, empirical Bayes differential expression, standardized cross-cohort evidence, and gene-set enrichment. Five discovery-informed modules formed an immune-resistance score. Conditional leave-one-out and leave-cohort-out analyses refitted normalization within training data and compared the fixed score with a nested ridge-logistic model. Survival, composition-adjustment, and prioritization sensitivity analyses were performed. Single-cell counts underwent quality control, unsupervised clustering, and patient-level pseudobulk analysis. MAL2 was investigated in A549 and H1299 cells using knockdown, growth, motility, apoptosis, HLA-ABC, cytokine, and PBMC co-culture assays.

**Results:**

No individual gene met FDR <0.05 in any of the three discovery contrasts. Favorable-response genes were enriched for adaptive immune processes, whereas the unfavorable list showed weaker DNA-replication enrichment. Conditional leave-cohort-out AUCs for the dual-axis score were 0.964, 0.859, and 0.773; performance did not consistently exceed the immune axis. In GSE190266, outcome analysis yielded a cohort-relative AUC of 0.617 with incomplete module coverage. Higher scores were associated with longer PFS in two evaluated cohorts. Quality control retained 91,762 single cells. MAL2 remained epithelial-enriched and ranked third among 58 evaluable candidates, but its patient-level epithelial response contrast and standalone PFS associations were not significant. Knockdown reduced growth and motility-related readouts, increased apoptosis and HLA-ABC expression, and altered cytokine and co-culture activation readouts.

**Conclusion:**

The immune-resistance framework links cohort-level response patterns to epithelial candidates for functional investigation. MAL2 depletion alters growth and immune-related phenotypes in lung cancer cells, supporting further investigation of its biological role.

## 1 Introduction

Lung cancer remains the leading cause of cancer-related death worldwide, and non-small cell lung cancer accounts for most cases encountered in clinical practice (Bray et al., 2024). Immune checkpoint blockade has changed this field more than almost any other therapeutic advance in the past decade. Anti-PD-1/PD-L1-based strategies improve survival in advanced disease and have also moved into perioperative settings, including neoadjuvant chemoimmunotherapy for resectable tumors (Reck et al., 2016; Gandhi et al., 2018; Hellmann et al., 2019; Forde et al., 2022). Even so, clinical benefit is uneven. Some patients achieve durable responses or major pathological regression, whereas others progress early despite apparently reasonable clinical indications for treatment. This heterogeneity makes biomarker development a practical necessity rather than a purely exploratory exercise.

The difficulty is that immunotherapy response is not governed by a single biological axis. PD-L1 expression and tumor mutational burden have clear clinical and biological relevance, but neither marker fully explains sensitivity or resistance (Rizvi et al., 2015; Ayers et al., 2017). A tumor may carry neoantigens but fail to present them efficiently. Another may contain cytotoxic lymphocytes but remain protected by tumor-cell programs that dampen antigen presentation, increase proliferative fitness, or reshape the local immune milieu. Studies of NSCLC and other cancers increasingly suggest that response to checkpoint blockade emerges from the interaction between tumor-extrinsic immune pressure and tumor-intrinsic escape mechanisms (Lau et al., 2022; McGranahan et al., 2017; Zaretsky et al., 2016; Liu et al., 2026). A useful biomarker strategy should therefore preserve both sides of this biology: the immune programs that favor response and the tumor-cell states that erode immune recognition.

Transcriptomic data offer one way to make this balance measurable. Bulk RNA-seq can quantify cytotoxic activity, antigen presentation, interferon-related inflammation, proliferation, and epithelial resistance programs across clinical cohorts. Single-cell RNA-seq then helps assign these signals to their cellular sources, separating lymphocyte- or myeloid-derived activity from tumor-lineage expression (He et al., 2024). In lung cancer, public immunotherapy datasets and single-cell studies have provided a valuable foundation for this type of integrative analysis, including cohorts treated with anti-PD-1 therapy, neoadjuvant PD-1 blockade combined with chemotherapy, and immune-panel profiling after checkpoint inhibition (Hu et al., 2023; Cho et al., 2020; Prat et al., 2017; Jung et al., 2019; Kim et al., 2020). These resources make it possible to move from response-associated transcriptional patterns to cell-type-aware target prioritization, which is particularly important when the final goal is experimental validation in tumor cells.

Here, we examined an immune-resistance dual-axis framework integrating cytotoxic T/NK activity, antigen/APC signaling, checkpoint-inflamed states, proliferation, and epithelial-associated resistance features. We evaluated the contribution of each axis, the sensitivity of candidate rankings, and the extent to which tissue composition explained the observed associations. Single-cell localization connected the cohort-level analyses to tumor-cell experiments, for which MAL2 was selected as a tractable epithelial-associated candidate. The experimental work examined cell growth, motility, apoptosis, HLA-ABC expression, cytokine readouts, and responses in PBMC-containing cultures.

## 2 Methods

### 2.1 Data sources and patient cohorts

Public expression matrices and clinical annotations were retrieved from the Gene Expression Omnibus (GEO) (Barrett et al., 2013). GSE126044 provided an anti-PD-1 response cohort (Cho et al., 2020), and GSE207422 provided pretreatment bulk profiles and a partly overlapping, mixed-timepoint single-cell sample set from neoadjuvant chemoimmunotherapy (Hu et al., 2023). GSE190265 supplied pretreatment expression profiles and PFS annotations, with GSE190266 providing a related expression resource for which 6-month outcome fields were retrieved from individual GEO sample records (Limagne et al., 2022). GSE135222 was used for PFS analysis (Jung et al., 2019; Kim et al., 2020). GSE93157 was reviewed as a mixed-cancer immune-panel reference (Prat et al., 2017); its limited feature coverage precluded full-score validation and it was excluded from the principal analyses.

### 2.2 Sample matching and analytical framework

The analysis included 16 profiles in GSE126044, 24 response-evaluable bulk profiles in GSE207422, 43 matched supplementary expression and clinical records in GSE190265, 27 profiles in GSE135222, and 70 profiles in GSE190266. The original publication describes 43 patients in the France #3 cohort (Limagne et al., 2022), consistent with the 43 unique identifiers shared by the GSE190265 supplementary expression and clinical files. The GEO series description instead lists 35 patients and registers 34 GSM records. Analyses used the published processed-file cohort; a sensitivity analysis was restricted to the 26 supplementary identifiers that matched registered GSM titles exactly. The GSE207422 single-cell resource contained three pretreatment biopsies and 12 post-treatment surgical samples from 15 patient identifiers; only P05, P07, and P08 overlapped with the bulk cohort.

Datasets were analyzed separately before comparing effect directions and module behavior. Treatment context, sampling time, and outcome definitions were retained rather than collapsed into a common clinical-response endpoint. The 43-record GSE190265 analysis and its registered subset were not considered independent validation cohorts.

### 2.3 Expression preprocessing and gene-level harmonization

GSE126044 integer counts were processed with edgeR (Robinson et al., 2010; Chen et al., 2025). Twenty-seven ambiguous date-like gene-symbol rows were excluded without attempting to infer their original identities. Low-expression genes were filtered with filterByExpr using the response design, normalization factors were estimated by the trimmed mean of M-values method (Robinson and Oshlack, 2010), and voom produced log2-CPM values and observation-level precision weights (Law et al., 2014). These weighted values were used for differential expression; unweighted voom expression values were used for module scoring. GSE135222, GSE190265, and GSE190266 TPM matrices were transformed as log2(TPM +1). The supplied log2-TPM values in GSE207422 were not log-transformed again.

Available gene identifiers were harmonized against gene symbols using the retained mapping resources and org.Hs.e.g.,.db. Ensembl version suffixes were removed before mapping where required. Duplicate rows in the processed non-count matrices were averaged after mapping; the retained GSE126044 count rows had unique symbols. Sample identities, expression distributions, and module coverage were inspected within each cohort. Quality-control fingerprints plotted the fraction of features with transformed expression greater than 1 against the expression interquartile range. PCA used the 2,000 most variable genes; separate sample-correlation heatmaps used Spearman correlations across the 2,500 most variable genes. Displayed PCA ellipses enclose an 80% group contour. No cross-cohort batch correction or pooled expression differential test was performed.

### 2.4 Clinical endpoint definition

GSE126044 compared five responders with 11 non-responders. GSE207422 compared nine MPR/pCR with 15 NMPR cases. In GSE190265, benefit was defined by no PFS event or PFS of at least 6 months. The discovery differential-expression contrast compared 14 benefit records with 19 events before 3 months; binary discrimination instead compared the same 14 benefit records with all 29 events before 6 months. No GSE190265 record was censored before 6 months. In GSE190266, expression identifiers matched 70 GSM titles exactly. Seventeen records were event-free at 6 months and 52 had an event by 6 months; one record censored at 0.03 months was excluded from the binary endpoint and retained as censored for survival analysis. PFS times were analyzed in the units supplied by each source, days for GSE135222 and months for the GSE190265/190266 resources.

### 2.5 Differential expression analysis

For each discovery contrast, a binary response design was fitted using lmFit and eBayes in limma (Ritchie et al., 2015). GSE126044 used voom precision weights; processed expression matrices used the limma-trend empirical Bayes option after retaining variable genes detected in at least three samples. The coefficient represented favorable minus unfavorable expression. Benjamini-Hochberg adjustment was applied over all genes tested in each contrast. Results were summarized at FDR thresholds of 0.05, 0.10, and 0.25; the last threshold was exploratory. Volcano colors denote nominal P < 0.05, with the FDR <0.05 gene count reported separately. The 43-record binary contrast and 26-record registered subset of GSE190265 were examined separately as sensitivity analyses.

### 2.6 Cross-cohort integration of response-associated genes

Cross-cohort integration was restricted to 15,568 genes tested in all three primary contrasts. For each gene and cohort, the favorable-minus-unfavorable difference was divided by the pooled within-group standard deviation and corrected for small-sample bias to obtain Hedges’ g. Approximate 95% intervals were calculated from its sampling variance. The sum of effect signs summarized concordance, and the unweighted mean standardized effect summarized magnitude without mixing raw fold-change scales.

For descriptive ranking, two-sided moderated P values were converted to normal deviates, assigned the sign of the corresponding effect, summed, and divided by the square root of three. This signed evidence score summarized directional support across the different treatment settings and endpoints. A separate sensitivity analysis varied the nominal-evidence coefficient in the initial raw-fold-change score from 0 to 0.50, including 0.15, and compared gene ranks and top-150 overlap. The initial score also defined the fixed candidate pool described in Section 2.15.

### 2.7 Functional enrichment analysis

GO Biological Process over-representation analysis used org.Hs.e.g.,.db and GO.db annotations (Gene Ontology Consortium, 2021). The 150 most strongly ranked concordant genes in each direction were mapped against the background of genes tested in all three contrasts. Hypergeometric tests were applied to Biological Process terms containing 10–500 background genes, and Benjamini-Hochberg adjustment covered every tested term within each directional analysis. The mapped background contained 13,717 genes. Query size, overlap, fold enrichment, nominal P, and adjusted P were retained for each term.

Enrichment plots displayed leading terms and their actual overlapping genes. Term-gene networks linked terms to query genes, and term-overlap networks connected pairs with a Jaccard similarity of at least 0.15 between their overlapping query-gene sets. Network layouts were calculated using igraph. Terms with adjusted P above 0.05 were retained as descriptive context. Shared membership was used to identify related terms rather than count them as independent biological processes.

### 2.8 Construction of immune and resistance modules

Five modules comprising 54 genes were assembled during the original discovery analysis from observed expression patterns and biological interpretation. This discovery-informed gene list defined the scoring framework. Cohort-relative analyses used detected module genes; conditional validation used the 50 genes shared by all three discovery cohorts, as detailed in Section 2.10. The cytotoxic T/NK module included CD8A, CD8B, GZMA, GZMB, GZMH, GZMK, PRF1, NKG7, CST7, CCL5, CXCL9, CXCL10, and IFNG. The antigen/APC module included HLA-DRA, HLA-DRB1, HLA-DPA1, HLA-DPB1, CD74, CTSS, LYZ, LST1, ITGAX, FCGR3A, and LILRA2. The checkpoint-inflamed module included PDCD1, CD274, PDCD1LG2, CTLA4, LAG3, TIGIT, HAVCR2, IDO1, and CXCL13. The proliferation-resistance module included MKI67, TOP2A, CCNB2, FANCI, PRC1, MCM2, MCM4, UBE2C, and BIRC5. The tumor-intrinsic resistance module included RAC3, ETV4, MEST, PYCR1, HOXC6, HOXC9, HOXC10, AKR1B10, MAL2, PKP1, DSP, and SCEL.

### 2.9 Dual-axis score construction

For each cohort, module scores were calculated as the mean z-scored expression of detected genes within each module. The immune-axis score was defined as the standardized sum of the cytotoxic T/NK module, antigen/APC module, and 0.5 times the checkpoint-inflamed module. The resistance-axis score was defined as the standardized sum of the proliferation-resistance and tumor-intrinsic resistance modules. The final dual-axis score was calculated as z(immune-axis score - resistance-axis score), so that higher values represented an immune-high and resistance-low state relative to the other samples in the same cohort. Gene-level and axis-level standardization parameters were estimated separately within each cohort. In held-out evaluations, gene means and standard deviations, and the subsequent immune-, resistance-, and combined-axis standardizations, were estimated from training samples only and applied unchanged to held-out samples. For descriptive visualization, samples were divided into within-cohort score tertiles or into quadrants defined by the signs of the two standardized axes. Tertile outcome fractions summarize an observed gradient rather than probability calibration.

### 2.10 Response prediction analysis

Apparent response AUCs were calculated within the three discovery cohorts using a rank-based formulation (Hanley and McNeil, 1982). Conditional leave-one-out and leave-cohort-out evaluations used the 50 module genes detected in all three cohorts; HLA-DRA, HLA-DRB1, HLA-DPA1, and HLA-DPB1 were absent from the GSE190265 matrix and were excluded from these evaluations. Gene-level and axis-level standardization parameters were fitted within each training split. Comparators were the immune axis, the sign-reversed resistance axis, and a ridge-logistic model using the five module scores. For ridge regression, C was selected from 0.01, 0.1, 1, 10, and 100 by threefold stratified inner cross-validation minimizing log loss; score normalization was refitted inside each inner split. Each outer split yielded held-out predictions. Performance estimates were conditional on the discovery-informed module selection (Varma and Simon, 2006). AUC intervals and paired AUC differences used 1,200 outcome-stratified bootstrap resamples of the fixed held-out predictions; precision-recall AUC was also retained.

### 2.11 Survival analysis

Kaplan-Meier curves used median-defined groups, with log-rank comparisons (Mantel, 1966). Cox models evaluated continuous score or standardized MAL2 expression, with age adjustment in GSE135222 and separate epithelial- or leukocyte-proxy sensitivity adjustments. Proportional hazards were assessed using Schoenfeld residuals. For MAL2 in GSE135222, where this assumption was violated, an exploratory time-varying coefficient model included MAL2 and MAL2 multiplied by log(time +0.1); its joint Wald test was retained. Multiple percentile-based groupings were examined only as exploratory cutoff sensitivity, not to select a clinical threshold.

### 2.12 Robustness and cutoff sensitivity analyses

All weighting and ablation analyses used the same nested score definition. The checkpoint coefficient was varied over 0, 0.25, 0.50, 0.75, and 1. Each module was separately multiplied by 0, 0.5, 1, or 2 before recalculating both axes and the final standardized difference. Resulting AUCs and Spearman rank correlations were compared with the full score. Composition sensitivity used disjoint transcript proxies: EPCAM, KRT8, KRT18, KRT19, KRT7, and MUC1 for epithelial content; PTPRC, CD3D, CD3E, TRAC, CD79A, MS4A1, and AIF1 for leukocyte content. Proxy scores were mean within-cohort gene z scores and were entered one at a time into response logistic models. They were not interpreted as absolute tumor purity or cell fractions.

GSE190266 transportability was assessed using the 6-month PFS-derived binary endpoint defined in Section 2.4. The primary cohort-relative analysis used 42 detected genes from the 54-gene module list. A secondary projection used the 50-gene common-feature framework and normalization parameters fitted in the three discovery cohorts. Of these 50 features, 38 were measured in GSE190266 and 12 were assigned their respective training means: PRF1, NKG7, PDCD1, PDCD1LG2, TIGIT, TOP2A, PRC1, UBE2C, RAC3, PYCR1, PKP1, and SCEL. The four HLA class II genes excluded from the training framework were also omitted from this projection. The cohort had previously contributed unlabeled expression summaries; its outcomes were retrieved for the transportability analysis.

### 2.13 Single-cell annotation and quality assessment

The complete GSE207422 UMI matrix was processed using Scanpy (Wolf et al., 2018). Cells were retained with 200–7,500 detected genes, at least 500 UMIs, and at most 20% mitochondrial counts. Scrublet was run separately for each sample with an expected doublet rate of 0.06 and an automatically estimated threshold (Wolock et al., 2019); predicted doublets were removed. Genes detected in fewer than three retained cells were excluded. Counts were preserved for pseudobulk analysis, while visualization used total-count normalization to 10,000 followed by log1p transformation. Three thousand highly variable genes were selected using the Seurat-v3 flavor with sample as the batch key. Principal components were calculated after scaling; a 20-neighbor graph used 30 PCs, followed by UMAP and Leiden clustering at resolution 0.5 (Traag et al., 2019). The seed was 20260907. Cluster markers were evaluated by Wilcoxon testing and canonical expression patterns. Strong CD3/TRAC expression distinguished cytotoxic T cells from NK-like cells; unresolved mixed-marker clusters were retained as such. Epithelial identity was marker-defined and was not equated with CNV-confirmed malignancy. No ambient-RNA correction was performed because empty-droplet profiles were unavailable.

### 2.14 Single-cell ecosystem and candidate localization analysis

Cell-type fractions were calculated separately for each sample using all retained cells as the denominator. Inferential response comparisons were restricted to the 12 post-treatment surgical samples, four MPR/pCR and eight NMPR; pretreatment biopsies were not pooled into this comparison. Two-sided patient-level Wilcoxon tests were adjusted across the tested cell types by the Benjamini-Hochberg method. Zero-recovery fractions were retained rather than dropping a patient from the denominator. Cell-fraction correlations and correlations between candidate all-cell expression and cell fractions were calculated across the same 12 post-treatment samples using Spearman coefficients; candidate-composition P values were BH-adjusted across cell types separately for each gene.

Candidate expression and detection fractions were first calculated within each patient and cell type, then summarized across patients with equal patient weighting among samples in which the corresponding cell type was recovered. A cell-type-specific expression value was undefined, rather than zero, when no cells of that type were recovered. Raw UMI counts were summed for each patient-by-cell-type combination containing at least 20 cells. Count-based differential expression required at least three patients per response group and used filterByExpr, TMM normalization, voom, and limma empirical Bayes modeling. Multiple testing was controlled within each cell-type contrast. The epithelial comparison included four MPR/pCR and eight NMPR patients. This patient-level inference avoids treating individual cells as independent biological replicates (Squair et al., 2021).

### 2.15 Candidate prioritization and MAL2-focused analysis

The 66-gene candidate pool comprised the 60 highest-ranked genes under the initial unfavorable-expression score plus six immune reference genes: NKG7, CCL5, PRF1, GZMH, CTSS, and LILRA2. The initial score was the negative sum of the three favorable-minus-unfavorable log-fold changes plus 0.15 times the sum of -log10(P), with P values bounded below at 1e-300. This pool was held fixed for reassessment using standardized unfavorable signed evidence, effect-direction consistency, presence across five bulk expression cohorts, patient-mean epithelial enrichment relative to immune compartments, and inverse Spearman association with the dual-axis score. Eight genes lacked single-cell expression summaries: HOXC9, HOXC6, CKMT1B, S100A7, CTAGE15, SPRR2E, SPRR2A, and CTAGE8. Complete-feature ranking therefore included 58 genes without imputation of localization features. Each candidate was removed from its module before recalculating the score used in its correlation feature. Epithelial enrichment was the difference between mean epithelial and immune-compartment expression summaries; epithelial detection fractions were reported separately.

Feature values were min-max scaled within the 58-gene complete-feature pool. Heuristic weights of 0.34, 0.18, 0.16, 0.16, and 0.16 were applied to the evidence, direction, presence, epithelial-enrichment, and inverse-correlation features, respectively. Sensitivity analyses used equal weights, no epithelial term, each feature omitted in turn, and 1,000 random positive weight vectors normalized to sum to one. Rank ranges and top-five frequencies summarized the dependence of candidate prioritization on these choices.

MAL2-focused analyses reported within-cohort differential expression, standardized effects, response AUCs for low MAL2 expression, and continuous and median-group PFS associations. Single-cell analyses distinguished its overall association with epithelial abundance from its expression difference within epithelial cells. PFS analyses were not included as an additional fitted component of the prioritization score. MAL2 correlations with modules were calculated within each bulk cohort. Correlations with selected lineage and immune markers were summarized by the arithmetic mean and minimum-to-maximum range across the three discovery cohorts; these ranges were not confidence intervals. The association with residual tumor fraction in GSE207422 was assessed by a two-sided Spearman test.

### 2.16 Cell lines and cell culture

Human bronchial epithelial BEAS-2B cells, human lung cancer cell lines A549, NCI-H1299, NCI-H1975, HCC827, NCI-H460, and human embryonic kidney HEK293T cells were used. BEAS-2B cells were maintained in bronchial epithelial cell culture medium, A549 cells in F-12K medium, NCI-H1299, NCI-H1975, HCC827, and NCI-H460 cells in RPMI-1640 medium, and HEK293T cells in high-glucose DMEM. Basal media were supplemented with 10% fetal bovine serum and 1% penicillin-streptomycin when applicable. Cells were maintained at 37 °C in a humidified incubator with 5% CO_2_, passaged at approximately 70%–80% confluence, and routinely tested to exclude *mycoplasma* contamination.

### 2.17 Generation of stable MAL2-knockdown cells

MAL2-knockdown lung cancer cells were generated using a lentiviral shRNA system. Two independent shRNAs targeting human MAL2 and a non-targeting control sequence were inserted into the pLKO.1-puro vector and designated sh-MAL2#1, sh-MAL2#2, and sh-NC. HEK293T packaging cells were co-transfected with the shRNA plasmid, psPAX2 packaging plasmid, and pMD2.G envelope plasmid using Lipofectamine 3000. Viral supernatants were collected at 48 and 72 h, centrifuged, filtered through a 0.45-μm filter, and used to infect lung cancer cells in the presence of polybrene. Stable knockdown populations were obtained by puromycin selection, and knockdown efficiency was validated by quantitative real-time PCR.

### 2.18 Quantitative real-time PCR

Total RNA was isolated using TRIzol reagent according to the manufacturer’s protocol. Qualified RNA samples were reverse-transcribed into cDNA using PrimeScript RT reagent Kit with gDNA Eraser, and quantitative real-time PCR was performed using TB Green Premix Ex Taq II on a QuantStudio 5 Real-Time PCR System. The amplification procedure consisted of 95 °C for 30 s, followed by 40 cycles of 95 °C for 5 s and 60 °C for 30 s. GAPDH was used as the endogenous control, and relative expression was analyzed using the 2^−ΔΔCt^ method (Livak and Schmittgen, 2001). Primer sequences were as follows: GAPDH-F, CTT​TTG​CGT​CGC​CAG​CCG; GAPDH-R, ATG​GAA​TTT​GCC​ATG​GGT​GGA​A; MAL2-F, GGA​GAT​TCT​GTT​CGG​GGG​TC; MAL2-R, ACA​TGC​CCA​GAA​AGA​GGA​GC; B2M-F, TGC​CGC​ATT​TGG​ATT​GGA​TG; B2M-R, CTC​TAA​GTT​GCC​AGC​CCT​CC; CXCL10-F, GTG​GAT​GTT​CTG​ACC​CTG​CT; CXCL10-R, GGA​GGA​TGG​CAG​TGG​AAG​TC; CCL5-F, CAG​TCG​TCT​TTG​TCA​CCC​GA; CCL5-R, GCC​TCC​CAA​GCT​AGG​ACA​AG; TGFB1-F, CGA​CTC​GCC​AGA​GTG​GTT​AT; TGFB1-R, GGT​AGT​GAA​CCC​GTT​GAT​GT.

### 2.19 CCK-8 and colony formation assays

Cell viability was evaluated using a Cell Counting Kit-8 assay. Lung cancer cells were harvested during logarithmic growth, seeded into 96-well plates in triplicate, and cultured under the indicated conditions. At each time point, 10 μL CCK-8 reagent was added to each well containing 100 μL medium, followed by incubation for 1–2 h protected from light. Absorbance at 450 nm was measured using a microplate reader, and OD450 values were plotted at 0, 24, 48, and 72 h.

Clonogenic capacity was assessed by colony formation assay. Cells were seeded into 6-well plates at low density and cultured for approximately 10–14 days until visible colonies formed. Colonies were washed with PBS, fixed with 4% paraformaldehyde, stained with crystal violet, air-dried, and photographed. The stained colony area was quantified using ImageJ (Schneider et al., 2012) and expressed as an area percentage.

### 2.20 Wound healing and transwell invasion assays

Cell migration was examined using a wound healing assay. Cells were seeded into 6-well plates and cultured until the monolayer reached approximately 90% confluence. A linear wound was generated with a sterile 200-μL pipette tip, detached cells were removed by PBS washing, and cells were cultured in serum-reduced medium. Images were captured at 0 h and 24 h using an inverted microscope. Wound area or width was measured using ImageJ, and the wound closure rate was calculated relative to the initial wound area.

Invasive capacity was determined using Matrigel-coated Transwell chambers with 8.0-μm pore membranes. Cells were resuspended in serum-free medium and seeded into the upper chamber, while complete medium containing 10% fetal bovine serum was added to the lower chamber. After incubation for 24–48 h, cells remaining on the upper surface were removed, and invaded cells on the lower membrane surface were fixed, stained with crystal violet, photographed, and quantified as stained invasion area using ImageJ.

### 2.21 Flow cytometry and immunofluorescence assays

Cell apoptosis was evaluated using APC Annexin V and propidium iodide staining. Cells were harvested together with floating cells, washed with cold PBS, resuspended in Annexin V binding buffer, stained for 15 min in the dark, and analyzed using a CytoFLEX flow cytometer. Data were processed using FlowJo v10 software, and apoptotic cells were defined as Annexin V-positive cells, including early and late apoptotic populations.

Cell-surface HLA-ABC expression was examined by flow cytometry using PE-Cyanine5-conjugated anti-human HLA-ABC antibody. Unstained or isotype controls were used to define the positive gate, and HLA-ABC expression was presented as the percentage of HLA-ABC-positive cells.

Immunofluorescence staining was performed to evaluate HLA-ABC expression and distribution. Cells grown on coverslips were fixed with 4% paraformaldehyde, permeabilized with 0.1% Triton X-100, blocked with 5% bovine serum albumin, incubated with anti-HLA-ABC primary antibody overnight at 4 °C, and then incubated with Alexa Fluor 488-conjugated secondary antibody. Nuclei were counterstained with DAPI. Images were captured under identical exposure settings, and mean fluorescence intensity was quantified using ImageJ.

### 2.22 ELISA and tumor cell-PBMC co-culture

Cytokines in culture supernatants were measured by ELISA. For tumor cell-derived cytokine detection, supernatants from stable MAL2-knockdown or control cells were collected after culture for the indicated time. For co-culture experiments, supernatants were harvested after tumor cells were co-cultured with PBMCs. Samples were centrifuged to remove debris and stored at −80 °C until analysis. Human IL-6, TGF-β1, IFN-γ, and TNF-α levels were measured using commercial ELISA kits according to the manufacturer’s protocols. For TGF-β1 measurement, samples were activated before detection as required. The plotted cytokine endpoint was relative signal normalized to the control reference.

For direct co-culture, cryopreserved human PBMCs purchased from STEMCELL Technologies (catalog no. 70025) were thawed, recovered in RPMI-1640 medium supplemented with 10% fetal bovine serum and 1% penicillin-streptomycin, and assessed by trypan blue viability staining. H1299 and A549 cells expressing sh-MAL2#1 or sh-NC were seeded and allowed to adhere overnight. PBMCs were then added directly to the tumor cell monolayer, with tumor cells cultured without PBMCs as non-co-culture controls. Co-cultures were maintained for 24–48 h before supernatant collection and cell harvesting.

### 2.23 Flow cytometric co-culture readouts

After co-culture, Annexin V/PI positivity was quantified within the scatter-gated population as the sum of early and late Annexin V-positive fractions. CD69 staining was evaluated in the recovered suspension-cell preparation and expressed as the percentage of CD69-positive gated events. These readouts were analyzed separately for the A549 and H1299 co-culture systems.

### 2.24 Statistical analysis

Analyses used R 4.5.2 and Python 3.11 with edgeR, limma, survival, Scanpy 1.11.5, scikit-learn, SciPy, and statsmodels, and GraphPad Prism 10.6.0 for experiments. Tests were two-sided; P < 0.05 and BH FDR <0.05 defined nominal and adjusted significance, respectively. Distribution comparisons used Wilcoxon tests, expression contrasts used limma or patient-pseudobulk models, and effect and AUC intervals were 95% intervals. Quantitative experiments were performed in three independent repeats (n = 3), with technical triplicates unless otherwise stated. The independent experiment was the unit of statistical analysis. Experimental data are summarized as mean ± SD. For qPCR, colony area, wound closure, invasion area, apoptosis, HLA-ABC, fluorescence-intensity, and ELISA endpoint comparisons, ordinary one-way ANOVA with Bonferroni–Šídák-adjusted selected pairwise comparisons was specified. CD69 fractions were compared by unpaired Welch’s t tests. ImageJ and FlowJo v10 were used for image and cytometry analysis. Plotting used R 4.5.1, ggplot2 4.0.2, patchwork 1.3.2, cowplot 1.2.0, ggridges 0.5.7, ggrepel 0.9.6, and igraph.

## 3 Results

### 3.1 Transcriptomic cohorts covered distinct treatment settings and endpoints

The bulk resources represented three distinct discovery settings: anti-PD-1 response in GSE126044, pathological regression after neoadjuvant chemoimmunotherapy in GSE207422, and PFS-defined benefit in GSE190265. GSE135222 contributed survival data, whereas GSE190266 provided an additional outcome assessment (Figures 1A,B). The matrices differed substantially in expression-record counts and feature coverage, with GSE93157 restricted to immune-panel reference use (Figure 1C). Coverage of the 54 module genes was complete in GSE126044 and GSE207422, but was 50/54 in GSE190265, 53/54 in GSE135222, and 42/54 in GSE190266 (Figure 1A). Expression-distribution fingerprints, cohort-specific PCA, and sample correlations documented within-cohort variation without pooling expression scales (Figures 1D–J). GSE190265 contained 43 matched supplementary expression-clinical records, of which 26 also matched registered GSM titles. Restricting the analysis to these 26 records retained a dual-axis apparent AUC of 0.806, compared with 0.793 in all 43 records.

**FIGURE 1:**
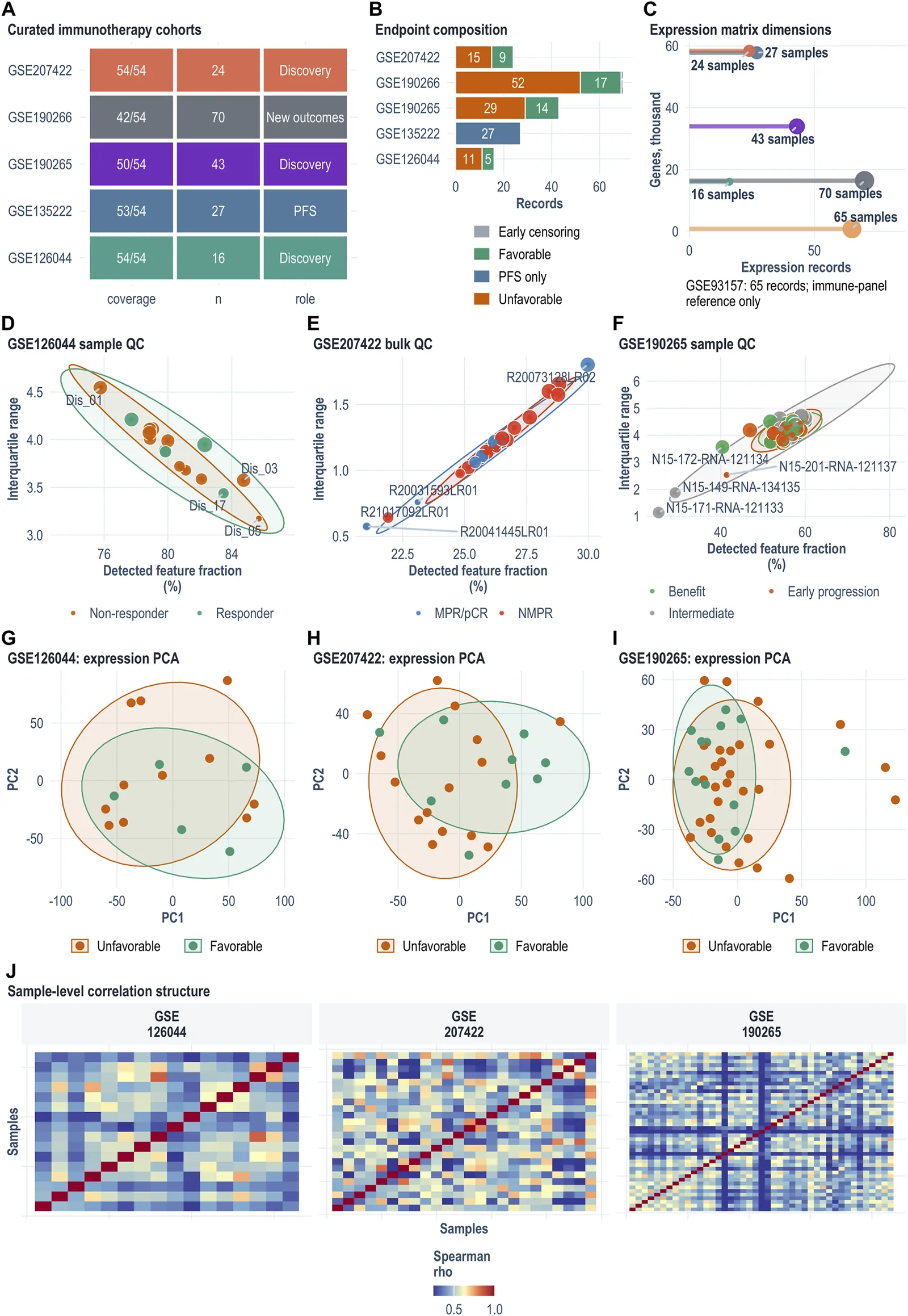
Cohort composition and expression quality assessment. **(A)** Cohort roles, expression-record counts, and coverage of the 54 module genes. **(B)** Binary outcome composition and survival-only records. **(C)** Expression-matrix dimensions; GSE93157 is an immune-panel reference. **(D–F)** Sample-level expression fingerprints for GSE126044, GSE207422, and GSE190265, plotting the fraction of features with transformed expression >1 against the interquartile range. **(G–I)** Corresponding cohort-specific PCA using 2,000 variable genes; ellipses show 80% group contours. **(J)** Sample Spearman correlations using 2,500 variable genes. Primary contrasts include 5 versus 11, 9 versus 15, and 14 versus 19 records, respectively. GSE190265 binary scoring uses 14 versus 29 records; GSE190266 has 69 binary-evaluable records and one early-censored record. GSE135222 contributes 27 survival records.

### 3.2 Cross-cohort response patterns were directional rather than uniformly significant

No gene passed FDR <0.05 in any primary discovery contrast (Figures 2A–C). GSE126044 retained 120 genes at FDR <0.10 and 608 at FDR <0.25; GSE207422 and the GSE190265 extreme-outcome contrast retained none at either threshold. Among 15,568 commonly tested genes, concordant directions were more restricted than nominal evidence in at least one cohort (Figure 2D). Pairwise Hedges’ g comparisons and their cohort-specific distributions showed that effect magnitudes were not interchangeable across datasets (Figures 2E,F). Signed-evidence ranking placed cytotoxic and lymphocyte-associated genes, including CST7, GZMH, PRF1, and GZMA, at the favorable end, while the unfavorable end contained a different set of response-associated candidates (Figures 2G,H). Gene-level evidence varied across cohorts even within these leading sets (Figure 2I). MAL2 fell within the concordant unfavorable region of the integrated effect map (Figure 2J), with its strongest evidence in GSE126044: log2 FC -3.44, nominal P = 0.00134, and FDR = 0.116. The cross-cohort signal was therefore more consistent at the level of effect direction and biological programs than at the level of individually replicated significant genes.

**FIGURE 2:**
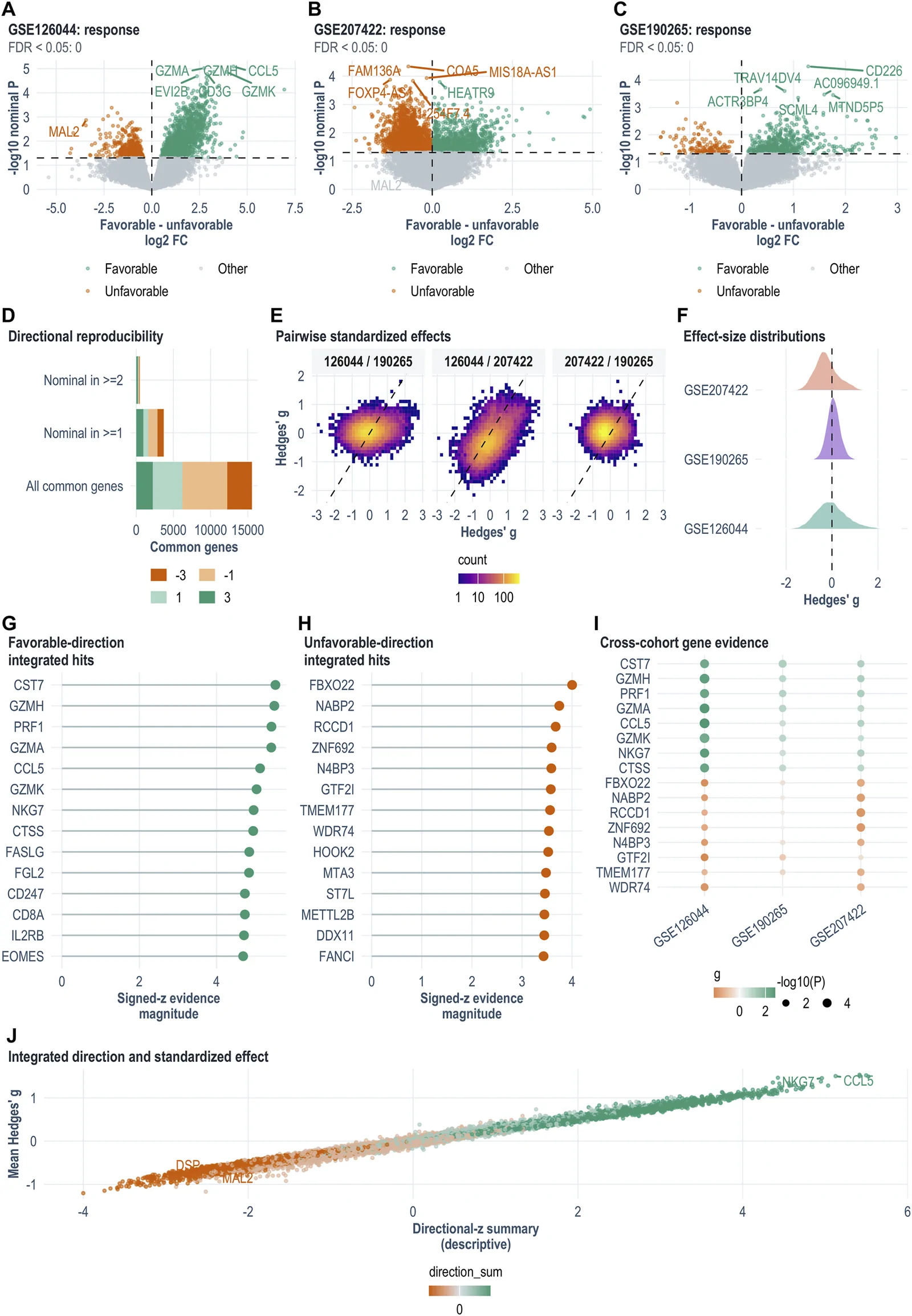
Differential expression and standardized cross-cohort evidence. **(A–C)** Favorable-minus-unfavorable expression contrasts for GSE126044, GSE207422, and GSE190265. Colors indicate nominal P < 0.05; dashed lines mark P = 0.05 and log2 FC = 0. The FDR <0.05 count is stated separately. **(D)** Effect-direction agreement among all common genes and subsets with nominal evidence in at least one or two cohorts. **(E)** Pairwise Hedges’ g distributions; bin color denotes gene count. **(F)** Cohort-specific standardized-effect densities. **(G,H)** Leading favorable and unfavorable signed-z evidence magnitudes. **(I)** Selected-gene effects and nominal evidence; size encodes -log10 P. **(J)** Signed-z summary versus mean Hedges’ g. Positive effects indicate greater expression in favorable groups. Integration includes 15,568 commonly tested genes and is descriptive, not a formal pooled-effect significance test.

### 3.3 Favorable-response enrichment was dominated by adaptive immune processes

The favorable-direction gene list was enriched for adaptive immune response, lymphocyte activation, and antigen-receptor signaling (Figure 3A). Adaptive immune response contained 41 query genes among 409 background-annotated genes and reached FDR = 9.46 × 10^−26^. Enrichment in the unfavorable list was weaker: DNA-templated DNA replication and DNA replication had FDRs of 0.00115 and 0.0180, respectively (Figures 3B,C). Term-gene networks localized these signals to overlapping immune or replication-associated genes, and the term-overlap map separated the two major groups of related processes (Figures 3D–F). At the sample level, immune-axis scores were higher in favorable groups in all three cohorts, whereas resistance-axis separation was weaker in GSE190265 (Figures 3G,H). The joint-axis distributions consequently varied by cohort rather than forming a universal response boundary (Figure 3I). Actual GO membership connected selected cytotoxic genes to the favorable processes (Figure 3J), linking the favorable expression pattern to immune processes.

**FIGURE 3:**
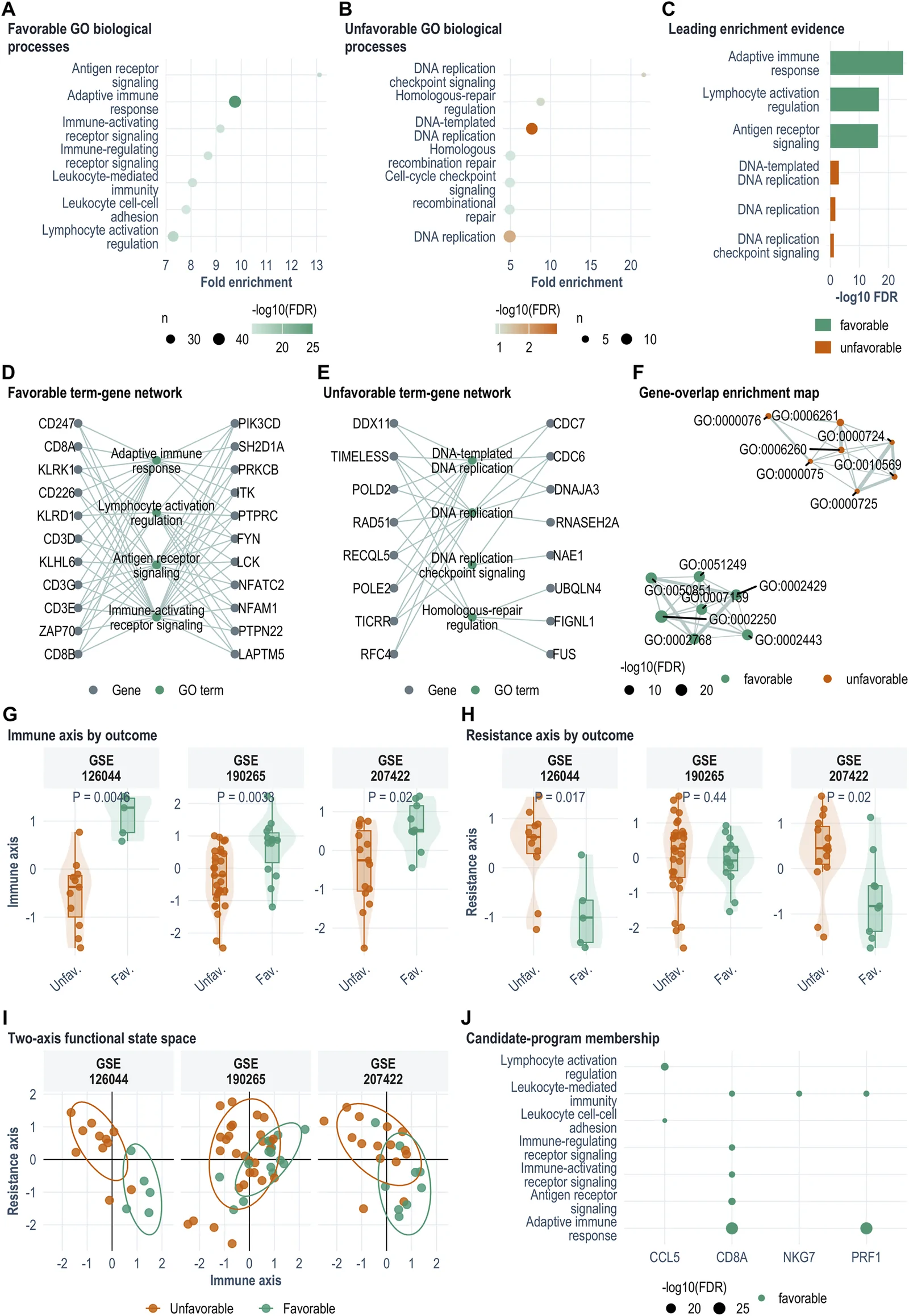
Response-associated Gene Ontology processes and sample-level axes. **(A,B)** Leading favorable- and unfavorable-direction GO Biological Process terms. Size denotes overlapping query genes and color denotes -log10 BH FDR. **(C)** Adjusted evidence for three leading terms per direction. **(D,E)** Term-gene membership networks. **(F)** Term-overlap network, with edges at Jaccard similarity ≥0.15. **(G,H)** Immune- and resistance-axis distributions by binary outcome. Points represent expression records; boxes show the median and interquartile range, whiskers extend to 1.5 times the interquartile range, and violins show density. Displayed P values are two-sided Wilcoxon tests. **(I)** Joint-axis states with 80% group contours. **(J)** Actual GO membership of selected cytotoxic genes. Enrichment used directional top-150 lists and a 13,717-gene mapped background; terms above FDR 0.05 are descriptive.

### 3.4 Module scoring translated response biology into sample-level immune and resistance states

The five modules showed unequal coverage and correlated, but nonidentical, sample-level behavior (Figures 4A–C). Their joint immune-resistance states overlapped across cohorts and outcomes (Figure 4D). Cytotoxic T/NK scores were higher in favorable groups, with Hedges’ g values of 2.50, 1.14, and 1.36 in GSE126044, GSE207422, and GSE190265, respectively (Figures 4E,J). Antigen/APC effects were positive but less clearly separated in GSE190265, while the checkpoint-inflamed module showed its weakest evidence in GSE207422 (Figures 4F,G). Proliferation and intrinsic-resistance scores were lower in favorable groups in GSE126044 and GSE207422; in GSE190265, the proliferation effect was close to zero and the intrinsic-resistance effect was modest (Figures 4H,I). Multiplicity-adjusted comparisons retained this uneven pattern (Figure 4J), indicating that the modules contributed different amounts of outcome-associated information in the three treatment settings.

**FIGURE 4:**
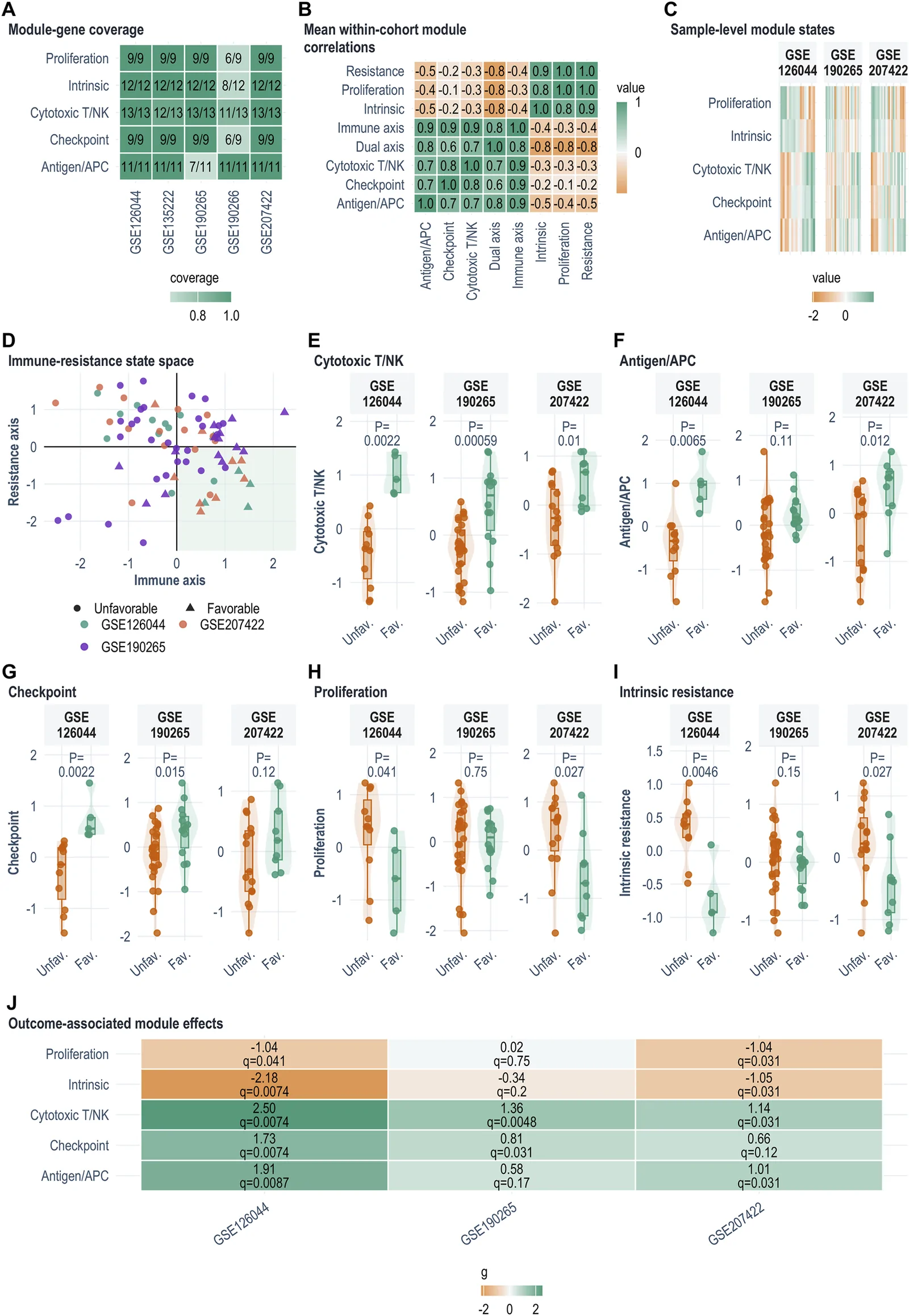
Coverage and outcome-associated behavior of the five expression modules. **(A)** Observed/configured gene counts per module. **(B)** Mean within-cohort Spearman correlation matrix across the three discovery cohorts. **(C)** Sample-level module scores ordered within cohort by outcome and dual-axis score. **(D)** Immune-resistance state space, with cohort colors and outcome shapes. **(E–I)** Cytotoxic T/NK, antigen/APC, checkpoint-inflamed, proliferation, and intrinsic-resistance module distributions. Points represent expression records, boxes show median and interquartile range with 1.5-interquartile-range whiskers, and violins show density; P values are nominal two-sided Wilcoxon tests. **(J)** Hedges’ g and BH-adjusted Wilcoxon P values, adjusted across eight module/axis/score comparisons per cohort. Binary groups contain 5 versus 11, 9 versus 15, and 14 versus 29 records in GSE126044, GSE207422, and GSE190265, respectively.

### 3.5 The dual-axis score combined immune and resistance states with a composition-related contribution

The score contrasted a standardized immune axis, including a checkpoint coefficient of 0.5, with a separately standardized resistance axis (Figure 5A). Module-score correlations and the geometry of the two axes confirmed that higher scores represented relatively greater immune activity and lower resistance-module expression (Figures 5B,C). Score distributions and record-level waterfalls showed substantial within-cohort variation, including outcome overlap (Figures 5D,E). Tertile profiles linked increasing scores to higher immune-module and lower resistance-module values, accompanied by a higher favorable-outcome fraction in the upper tertile (Figures 5F,G). Direct group comparisons and sign-defined quadrants showed the same score gradient (Figures 5H,I). Composition sensitivity, however, qualified this association. In GSE126044, the unadjusted response OR was 24.93 per SD score; after leukocyte-proxy adjustment, it was 6.22 with a wide 95% CI of 0.066–588.11 (P = 0.431). Associations in GSE207422 and GSE190265 remained positive after separate epithelial- or leukocyte-proxy adjustment, again with broad intervals (Figure 5J). Thus, part of the score variation tracked tissue composition, and its additional contribution differed across cohorts.

**FIGURE 5:**
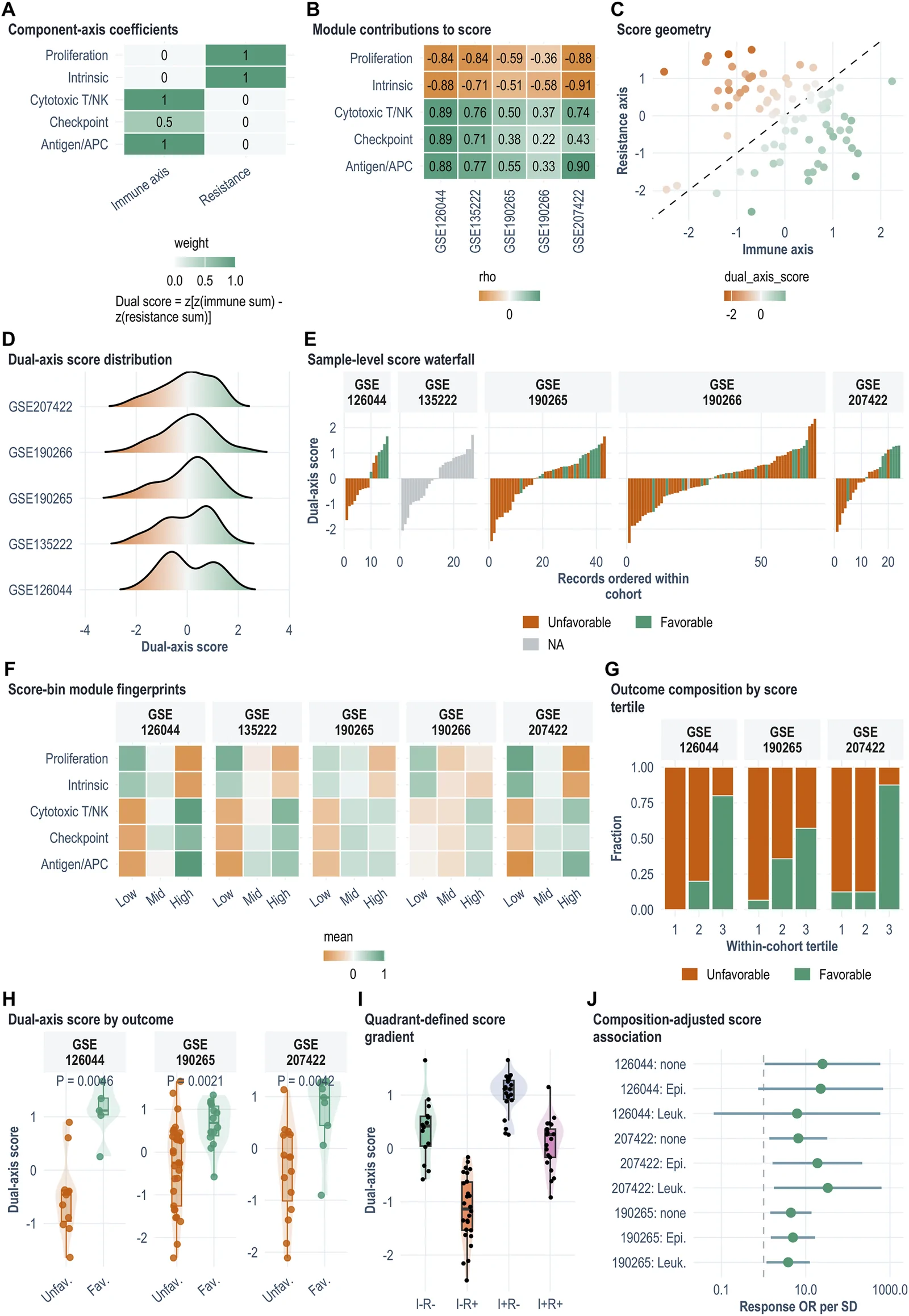
Construction and composition sensitivity of the dual-axis score. **(A)** Module coefficients before separate axis standardization. **(B)** Within-cohort module-score Spearman correlations. **(C)** Dual-axis scores in immune-resistance space. **(D)** Within-cohort score densities. **(E)** Record-level score waterfall; unavailable binary outcomes are gray. **(F)** Mean module values by within-cohort score tertile. **(G)** Outcome proportions by tertile in discovery cohorts. **(H)** Score distributions by binary outcome, with nominal two-sided Wilcoxon P values; boxes show median and interquartile range with 1.5-interquartile-range whiskers. **(I)** Score distributions across sign-defined axis quadrants; I and R denote immune and resistance axes. **(J)** Logistic-response ORs and 95% CIs per SD score, without adjustment or with separate epithelial- or leukocyte-transcript-proxy adjustment. Tertile proportions are observed outcomes, not probability-calibration estimates.

### 3.6 The dual-axis score was associated with response and PFS across the evaluated cohorts

Apparent dual-axis AUCs were 0.964 in GSE126044, 0.859 in GSE207422, and 0.793 in the 43-record GSE190265 binary comparison (Figures 6A–C). Bootstrap intervals were wide, and immune-axis discrimination was similar in several comparisons (Figure 6D). Higher median-defined scores were associated with longer PFS in GSE190265 and GSE135222 (log-rank P = 0.0041 and 0.015, respectively; Figures 6E,F). Continuous-score models gave an HR of 0.603 per SD in GSE190265 (95% CI 0.434-0.837, P = 0.00252) and an age-adjusted HR of 0.611 in GSE135222 (95% CI 0.399-0.935, P = 0.0232; Figure 6G), without evidence of a proportional-hazards violation for these score models. Observed event and censoring times and favorable-outcome fractions across tertiles illustrated the score-outcome gradient (Figures 6H,I). Conditional leave-cohort-out estimates were close to the apparent estimates for the dual-axis score, with a modest decrease in GSE190265 (Figure 6J).

**FIGURE 6:**
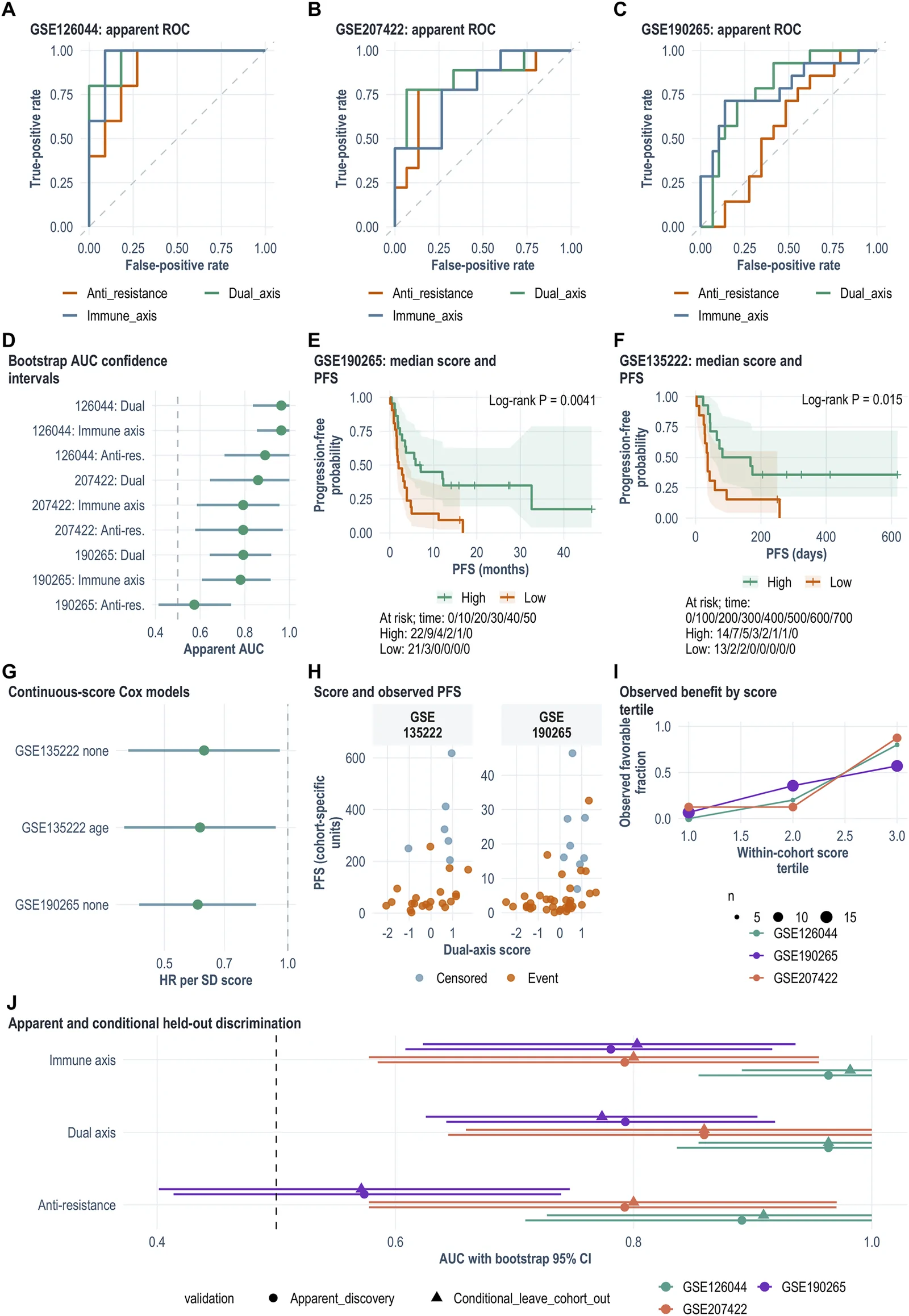
Apparent response discrimination and progression-free survival. **(A–C)** Apparent response ROC curves for the combined score, immune axis, and sign-reversed resistance axis in GSE126044, GSE207422, and GSE190265. **(D)** Corresponding AUCs with bootstrap 95% intervals. **(E,F)** Median-stratified score PFS in GSE190265 (43 records) and GSE135222 (27 records), with censoring marks, numbers at risk, log-rank P values, and 95% survival intervals. **(G)** Continuous-score Cox HRs per SD with 95% CIs; age adjustment is indicated. **(H)** Observed PFS and censoring against score. Time is in months for GSE190265 and days for GSE135222. **(I)** Favorable-outcome fractions by within-cohort score tertile; point size is record count. **(J)** Apparent and conditional leave-cohort-out AUCs with bootstrap 95% intervals. Discovery-cohort response estimates use 16, 24, and 43 records, respectively.

### 3.7 Held-out analyses showed variable performance and limited transportability

Using the 50 shared module genes, conditional leave-cohort-out AUCs for the dual-axis score were 0.964, 0.859, and 0.773 in GSE126044, GSE207422, and GSE190265, respectively; leave-one-out AUCs were 0.964, 0.852, and 0.793 (Figures 7A,B). The immune axis and five-module ridge model were competitive, and paired bootstrap differences did not show consistent dual-axis superiority (Figure 7C). Removing individual modules changed discrimination to different degrees, while sample-score ranks remained strongly correlated with the full score (Figures 7D,E). Comparison of the two conditional validation schemes also showed greater variation for some fitted ridge models than for the fixed score (Figure 7F). Varying the checkpoint coefficient had little effect on apparent AUC (Figure 7G). Across the tested PFS cutoffs, high-versus-low HRs remained below 1, although uncertainty varied; these multiple-threshold comparisons were exploratory (Figures 7H,I). In 69 evaluable GSE190266 records, the cohort-relative score based on 42 available module genes achieved an AUC of 0.617 (95% bootstrap interval 0.463-0.757). Projection of the 50-gene training framework, using 38 measured genes and 12 training-mean imputations, yielded an AUC of 0.661, compared with 0.675 for the immune axis and 0.618 for ridge regression (Figure 7J). In a separate 6-month PFS analysis of all 70 records, the score association was nonsignificant (HR 0.898, 95% CI 0.676-1.194, P = 0.460), with a PH violation (P = 0.00435). This additional cohort therefore provided weaker support for transportability than the discovery-cohort estimates suggested.

**FIGURE 7:**
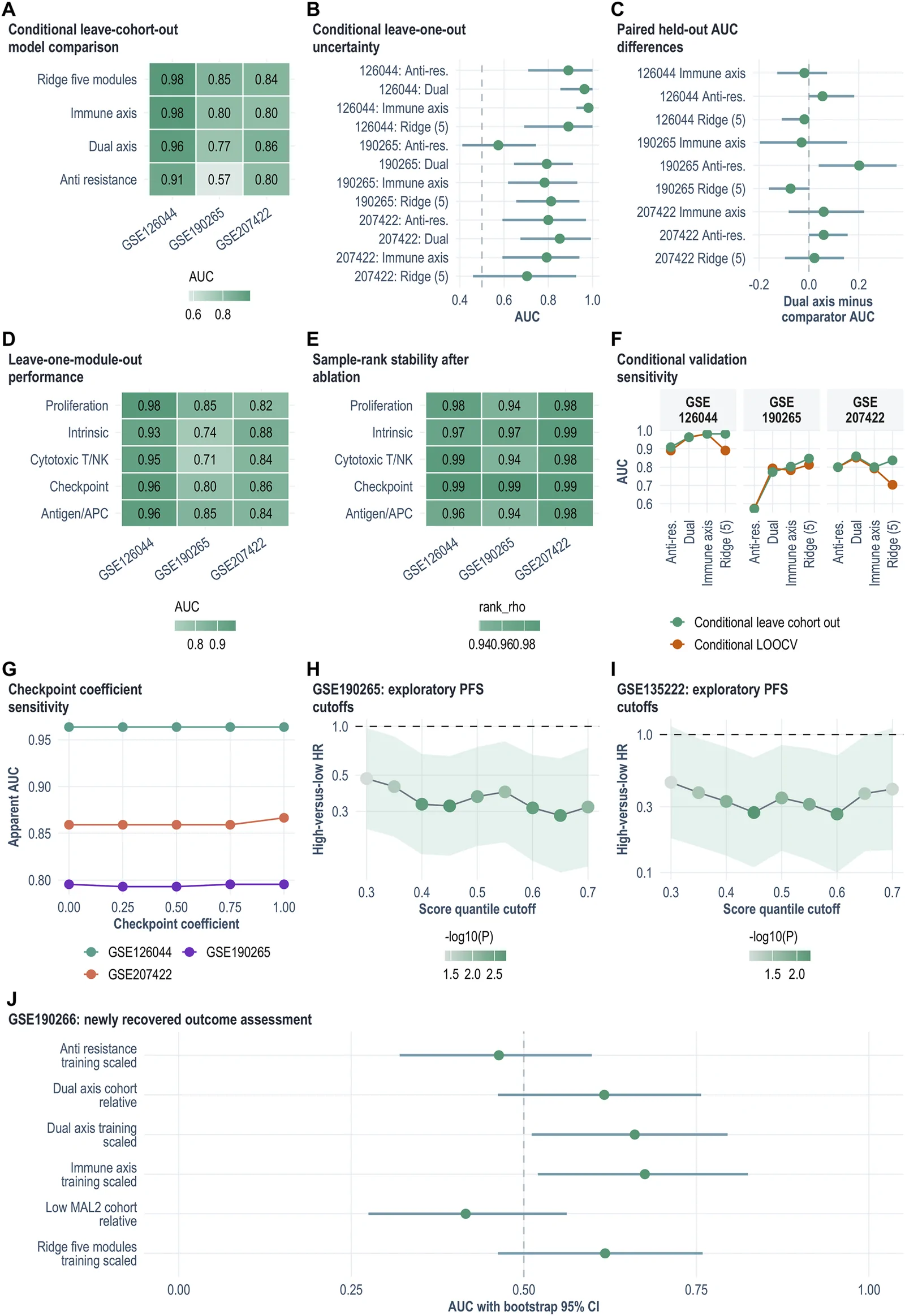
Conditional validation and score sensitivity. **(A)** Conditional leave-cohort-out AUCs for the score, component axes, and five-module ridge-logistic model. **(B)** Conditional leave-one-out AUCs with bootstrap 95% intervals. **(C)** Paired leave-cohort-out AUC differences, dual axis minus comparator, with 95% intervals. **(D,E)** Apparent AUC and sample-rank correlation after individual-module omission. **(F)** Comparison of conditional validation schemes. **(G)** Apparent AUC across checkpoint coefficients. **(H,I)** Exploratory high-versus-low score PFS HRs across cutoffs in GSE190265 and GSE135222; ribbons show 95% CIs and colors encode nominal P values. **(J)** GSE190266 AUCs with 95% intervals from 69 evaluable outcomes. **(A–C)** and F use the 50 module genes shared by the discovery cohorts, with discovery-informed membership and training-fitted normalization. Validation intervals use 1,200 outcome-stratified resamples of fixed predictions. In **(J)** cohort-relative scoring uses 42/54 genes; training-scaled projection uses 38 measured genes and 12 training-mean imputations within the 50-gene training framework.

### 3.8 Integrated prioritization retained MAL2 as an epithelial-associated candidate

Of the original 66 candidates, 58 had complete features for reassessment. DSP ranked first, CKS1B second, and MAL2 third under the original feature weights (Figure 8A). Cross-cohort effect profiles and placement in the integrated evidence distribution showed variable magnitudes even among genes with concordant unfavorable directions (Figures 8B,C). Inverse candidate-score correlations remained after removing each candidate from its own score module (Figure 8D), but the expression effects and MAL2 group distributions again showed that its strongest response association came from GSE126044 (Figures 8E,F). MAL2 rank ranged from 1 to 23 across weighting scenarios, with a median of 3 and top-five placement in 68.8% of scenarios (Figure 8G). Patient-averaged localization distinguished epithelial-enriched candidates from genes expressed more broadly across lineages (Figure 8H). MAL2 had an epithelial detection fraction of 0.717 and favorable localization for cell-line assays (Figure 8I). The shortlist evidence matrix illustrated the balance among directional evidence, expression localization, detectability, and score association (Figure 8J). MAL2 thus combined epithelial localization with consistently high, although weight-dependent, prioritization.

**FIGURE 8:**
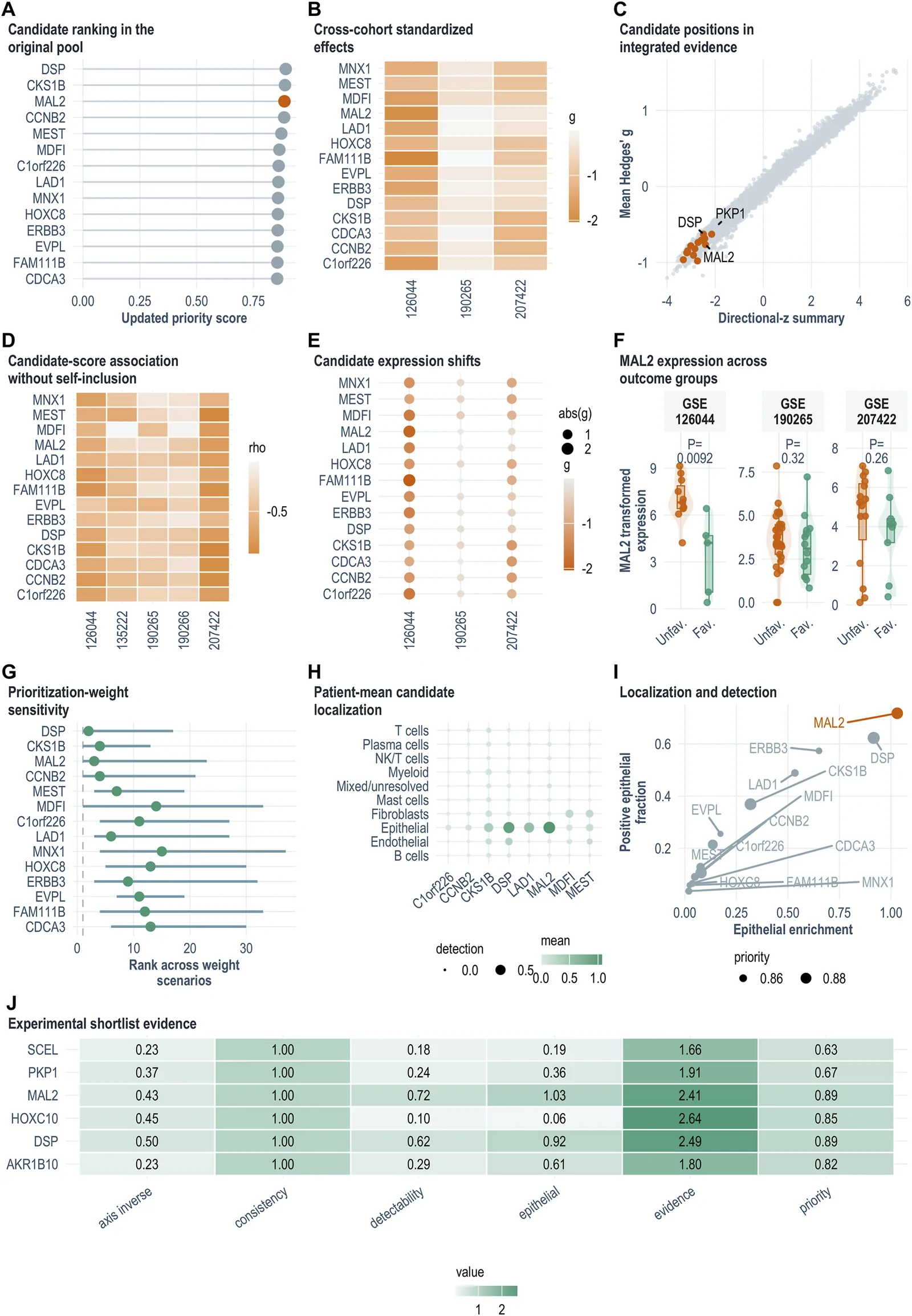
Candidate prioritization, weighting dependence, and cellular localization. **(A)** Leading candidate ranks; 58 of the original 66 candidates had complete features, with MAL2 ranked third. **(B)** Cross-cohort Hedges’' g. **(C)** Candidates within the full tested-gene evidence distribution. **(D)** Candidate-score Spearman correlations after candidate removal from its own module. **(E)** Candidate effects, with size encoding absolute Hedges’ g. **(F)** MAL2 expression by binary outcome with nominal Wilcoxon P values; boxes show median and interquartile range with 1.5-interquartile-range whiskers. **(G)** Median ranks and minimum-to-maximum ranges across 1,008 weighting scenarios. **(H)** Patient-averaged lineage expression and detection fraction for selected candidates. **(I)** Epithelial enrichment and detection fraction, with size encoding priority. **(J)** Quantitative evidence components for the six-gene experimental shortlist. Localization summaries weight patients equally within recovered compartments; the columns in J represent different measurement scales.

### 3.9 Single-cell ecosystem analysis contextualized the bulk response programs

Single-cell processing retained 91,762 cells from 15 samples, with T cells and myeloid cells forming the largest compartments (Figure 9A). Canonical-marker review of 27 expression clusters supported major lineage assignments, with unresolved mixtures retained explicitly (Figure 9B). Doublet exclusion, per-sample recovery, and cell-type fractions documented uneven representation across samples (Figures 9C–E). Pathological-response comparisons were restricted to four MPR/pCR and eight NMPR post-treatment patients. Mean compositions differed descriptively between these groups (Figure 9F), but none of the ten patient-level cell-fraction comparisons survived BH adjustment (Figure 9G). T-cell and NK/T-cell fractions tended to be higher in favorable cases, whereas epithelial fractions were highly variable (Figure 9H). The unsupervised embedding placed the annotated compartments in distinct expression regions, and post-treatment fraction correlations documented their compositional relationships (Figures 9I,J). The atlas resolved the cellular sources of the bulk-derived programs.

**FIGURE 9:**
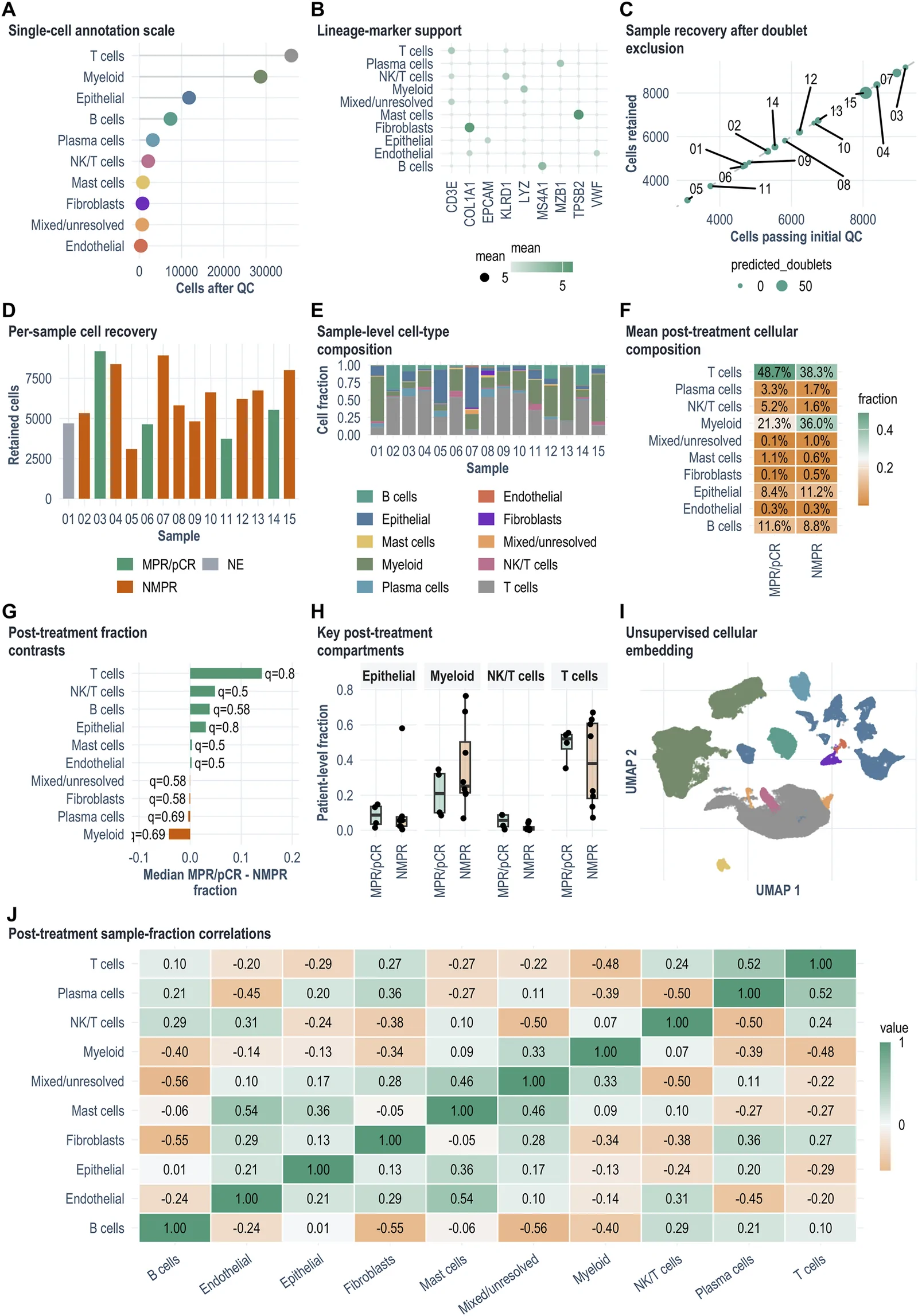
Single-cell annotation and post-treatment cellular composition. **(A)** Annotated cell-type counts among 91,762 retained cells. **(B)** Canonical lineage-marker expression averaged over annotated clusters; point size and color encode expression. **(C)** Cells retained before and after sample-wise doublet exclusion. **(D)** Per-sample recovery, with pretreatment samples labeled NE. **(E)** Sample-level cellular fractions. **(F)** Mean fractions in four MPR/pCR and eight NMPR post-treatment patients. **(G)** Patient-level median fraction differences and BH-adjusted two-sided Wilcoxon P values across 10 cell types. **(H)** Individual fractions for selected compartments, with median/interquartile-range boxes and 1.5-interquartile-range whiskers. **(I)** Unsupervised UMAP colored by lineage, using the key in E. **(J)** Post-treatment sample-fraction Spearman correlations. Fraction denominators include all retained cells per sample; pretreatment biopsies are excluded from response inference. NE, not evaluated for pathological response.

### 3.10 Single-cell target localization placed MAL2 within marker-defined epithelial compartments

Patient-averaged expression and epithelial-enrichment summaries localized MAL2 primarily to marker-defined epithelium (Figures 10A,B). Candidate pseudobulk contrasts across eligible compartments showed little adjusted evidence for response-associated changes (Figure 10C). MAL2 expression remained predominantly epithelial in both post-treatment response groups (Figure 10D), and the patient-level epithelial comparison was nonsignificant (Wilcoxon P = 1.00; Figure 10E). Detection fractions varied across samples in which epithelial cells were recovered (Figure 10F). By contrast, all-cell MAL2 abundance was strongly related to epithelial fraction across the 12 post-treatment samples (Spearman rho = 0.923, nominal P = 1.86 × 10^−5^; Figure 10G). Candidate-composition correlations extended this context to the other shortlisted genes (Figure 10H). The epithelial count-based pseudobulk model, using four favorable and eight unfavorable patients, gave a MAL2 log2 FC of −0.274, P = 0.552, and FDR = 0.856 (Figure 10I). Its high rank and epithelial detectability therefore coexisted with weak evidence for a response-associated change within epithelium (Figure 10J), favoring a composition-related explanation for part of its bulk signal.

**FIGURE 10:**
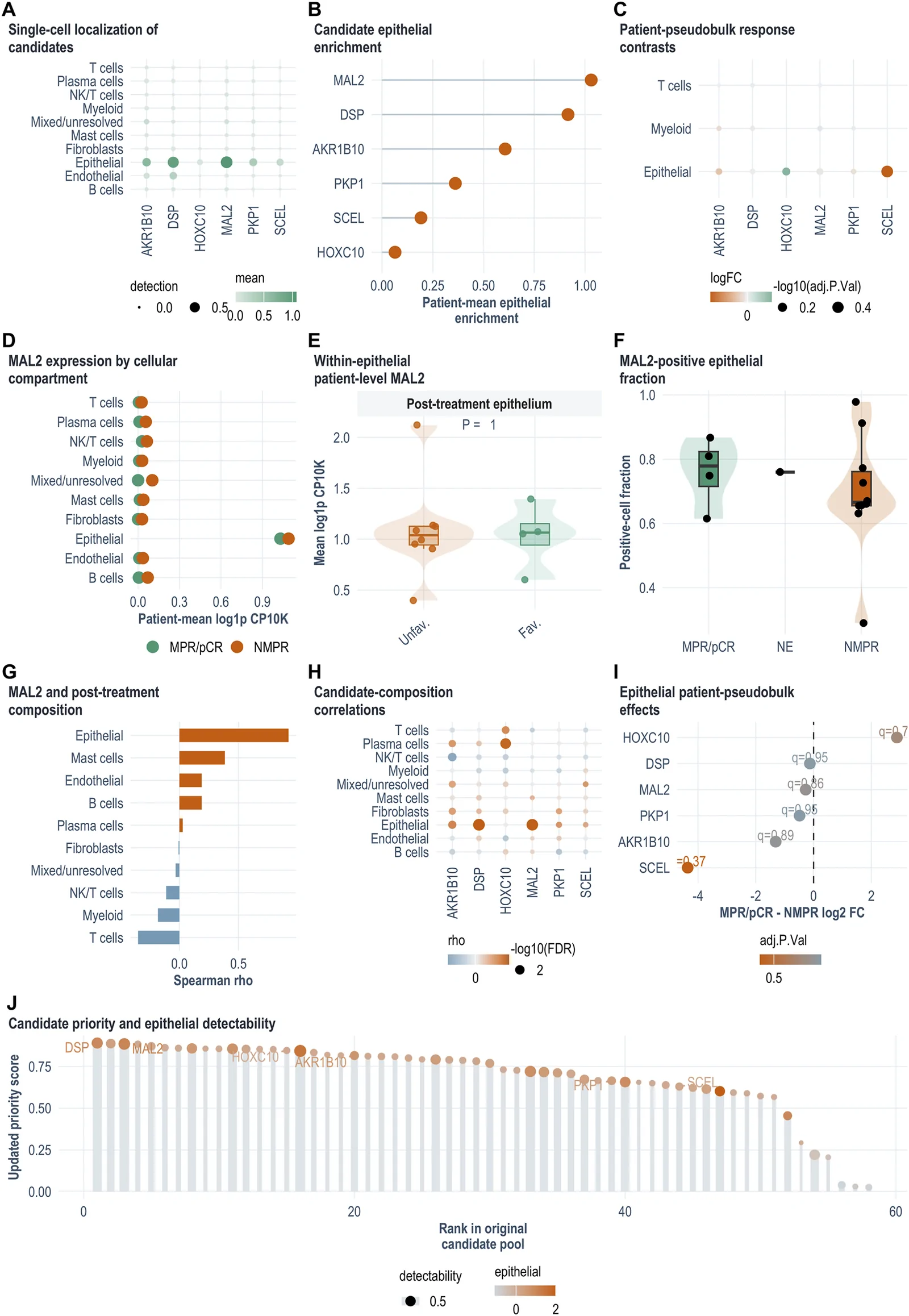
Candidate localization and within-epithelial MAL2 analysis. **(A)** Patient-averaged candidate expression by lineage; size denotes positive-cell fraction. **(B)** Mean epithelial-minus-immune expression enrichment. **(C)** Patient-pseudobulk response contrasts for candidates passing count filtering in eligible compartments; size denotes -log10 BH-adjusted P. **(D)** Post-treatment patient-mean MAL2 expression by lineage and response. **(E)** Individual epithelial MAL2 means, median/interquartile-range boxes, density, and nominal Wilcoxon P. **(F)** Positive epithelial fractions among samples with recovered epithelium; NE is pretreatment. **(G,H)** Candidate all-cell expression versus cell-fraction Spearman correlations in 12 post-treatment samples; H encodes within-gene BH-adjusted P by size. **(I)** Epithelial count-model effects and FDRs, using four favorable and eight unfavorable patients. **(J)** Ranking of 58 complete-feature candidates with epithelial enrichment and detectability. Expression is log1p counts per 10,000; a sample without recovered epithelium has no epithelial expression/detection estimate.

### 3.11 MAL2 carried response-related information but was not a standalone PFS biomarker

MAL2 standardized effects were −1.93, −0.304, and −0.064 in the GSE126044, GSE207422, and GSE190265 primary contrasts, respectively (Figure 11A). Outcome-stratified expression distributions and apparent ROC curves showed the same attenuation outside GSE126044 (Figures 11B,C). In GSE207422, the association with residual tumor fraction was weak and nonsignificant (Spearman rho = 0.25, P = 0.23; Figure 11D). Median MAL2 groups did not significantly separate PFS in GSE190265 or GSE135222 (Figures 11E,F). Continuous MAL2 models were also nonsignificant; GSE135222 additionally violated proportional hazards (P = 0.0377), limiting interpretation of its constant HR (Figure 11G). An exploratory time-varying model had a joint Wald P of 0.117. MAL2-module correlations were stronger for epithelial-associated resistance programs, while marker correlations varied across cohorts, with the displayed ranges representing cross-cohort variation rather than confidence intervals (Figures 11H,I). Low MAL2 yielded apparent AUCs of 0.927, 0.644, and 0.596 in the three discovery cohorts, but an AUC of 0.416 with newly retrieved GSE190266 outcomes (Figure 11J). MAL2 thus remained an epithelial-associated functional candidate rather than a reproducible standalone response or PFS biomarker.

**FIGURE 11:**
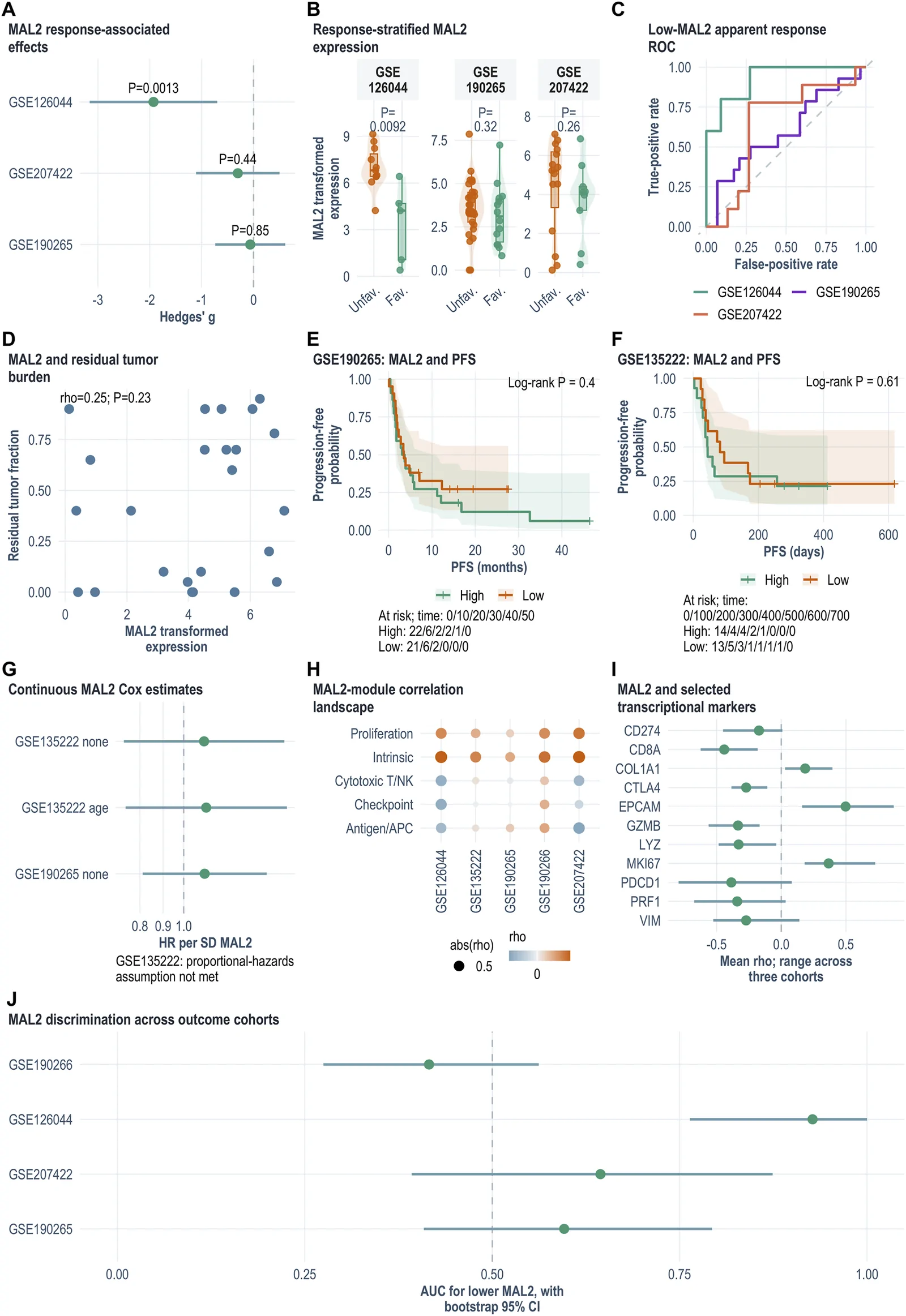
MAL2-focused expression, response, and survival analyses. **(A)** MAL2 Hedges’ g with approximate 95% intervals and nominal limma P values from the three primary contrasts. **(B)** MAL2 expression by binary outcome with nominal Wilcoxon P values, median/interquartile-range boxes, and 1.5-interquartile-range whiskers. **(C)** Low-MAL2 apparent ROC curves. **(D)** MAL2 and residual tumor fraction in GSE207422, with nominal Spearman statistics. **(E,F)** Median-stratified MAL2 PFS in GSE190265 and GSE135222, with censoring, risk counts, log-rank tests, and 95% survival intervals. **(G)** Continuous MAL2 Cox HRs per SD and 95% CIs; the GSE135222 PH assumption is violated. **(H)** Within-cohort MAL2-module Spearman correlations. **(I)** Mean MAL2-marker correlations across three discovery cohorts; whiskers are minimum-to-maximum ranges, not CIs. **(J)** Low-MAL2 AUCs and bootstrap 95% intervals, including 69 newly evaluated GSE190266 outcomes. GSE190265 uses 14 versus 19 records in A and 14 versus 29 in **(B,C,J)**.

### 3.12 MAL2 knockdown suppressed lung cancer cell proliferation and clonogenic growth

The epithelial enrichment and detectability of MAL2 motivated its functional evaluation in lung cancer cells. MAL2 expression was higher in lung cancer cell lines than in normal bronchial epithelial BEAS-2B cells, with A549 and H1299 cells showing the highest expression and therefore selected for subsequent assays (Figure 12A). Two independent shRNAs efficiently reduced MAL2 mRNA expression in both H1299 and A549 cells (Figures 12B,C). Functionally, MAL2 depletion reduced growth-associated CCK-8 absorbance over 72 h (Figures 12D,E). Consistent with this growth-inhibitory effect, colony formation was substantially reduced after MAL2 knockdown in both cell lines, as shown by representative colony images and quantitative colony-area analysis (Figures 12F–H). These results indicate that MAL2 supports proliferative and clonogenic capacity in lung cancer cells.

**FIGURE 12:**
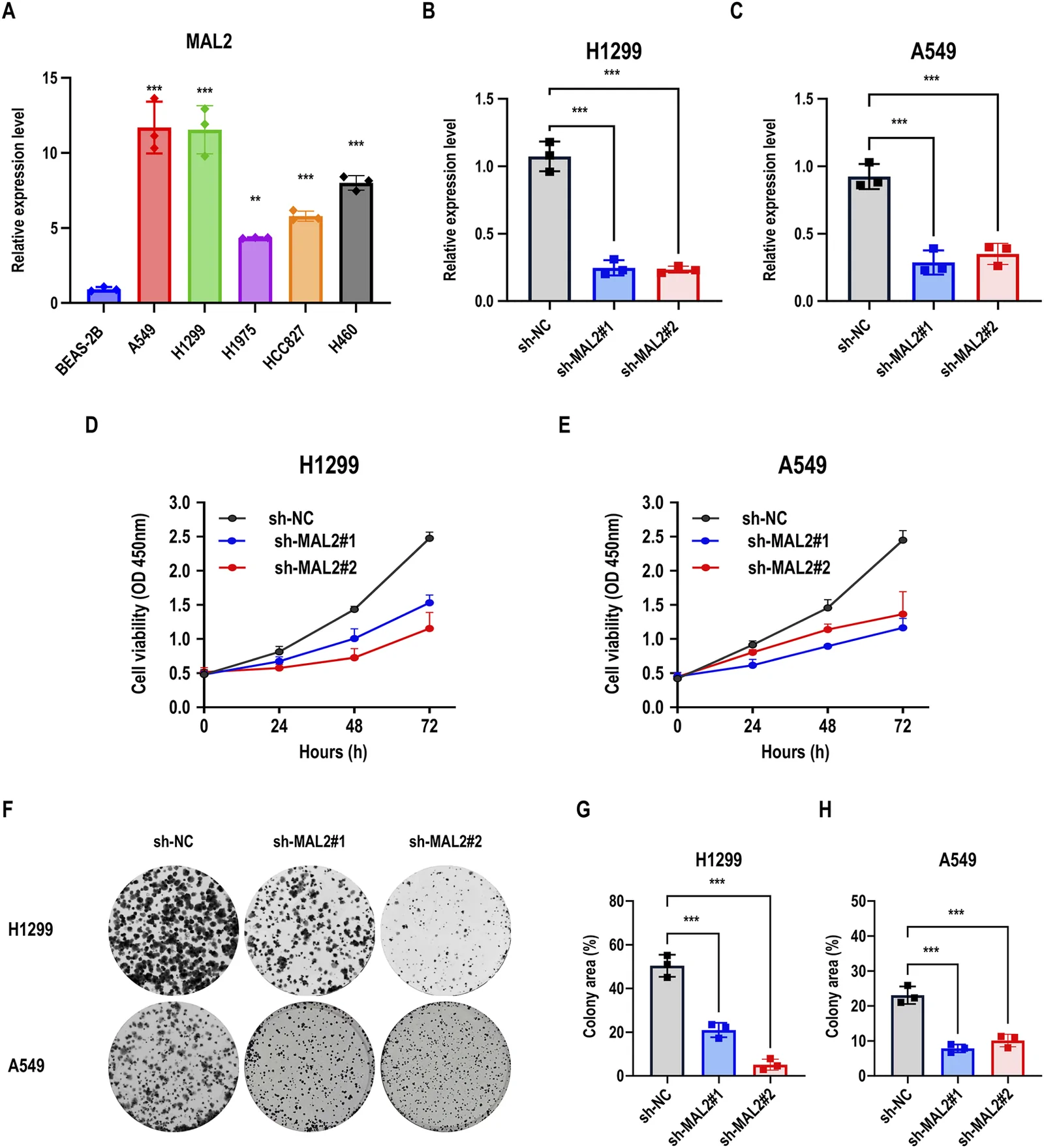
MAL2 knockdown suppresses proliferation and clonogenic growth in lung cancer cells. **(A)** MAL2 expression in normal bronchial epithelial BEAS-2B cells and lung cancer cell lines A549, H1299, H1975, HCC827, and H460. **(B,C)** qPCR validation of MAL2 knockdown efficiency in H1299 and A549 cells transduced with sh-NC, sh-MAL2#1, or sh-MAL2#2. **(D,E)** CCK-8 analysis of cell proliferation over 72 h. **(F)** Representative colony formation images in H1299 and A549 cells. **(G,H)** Quantification of colony area. Data are presented as mean ± SD from three independent experiments (n = 3). **(A–C)** and **(G,H)** used one-way ANOVA with Bonferroni–Šídák-adjusted comparisons. **P < 0.01, ***P < 0.001. In A, comparisons are versus BEAS-2B; other comparisons are indicated by brackets.

### 3.13 MAL2 depletion impaired migration and invasion while increasing apoptosis

We next examined whether MAL2 also influenced motility, invasion, and cell survival. In wound healing assays, MAL2-knockdown H1299 and A549 cells showed slower wound closure at 24 h than sh-NC controls (Figures 13A–C). Matrigel-coated Transwell assays further showed a pronounced reduction in invasive area after MAL2 silencing in both cell lines (Figures 13D–F). Flow cytometric Annexin V/PI staining then demonstrated that MAL2 knockdown increased the proportion of apoptotic cells, with both sh-MAL2#1 and sh-MAL2#2 producing higher apoptosis than the control group (Figures 13G–I).

**FIGURE 13:**
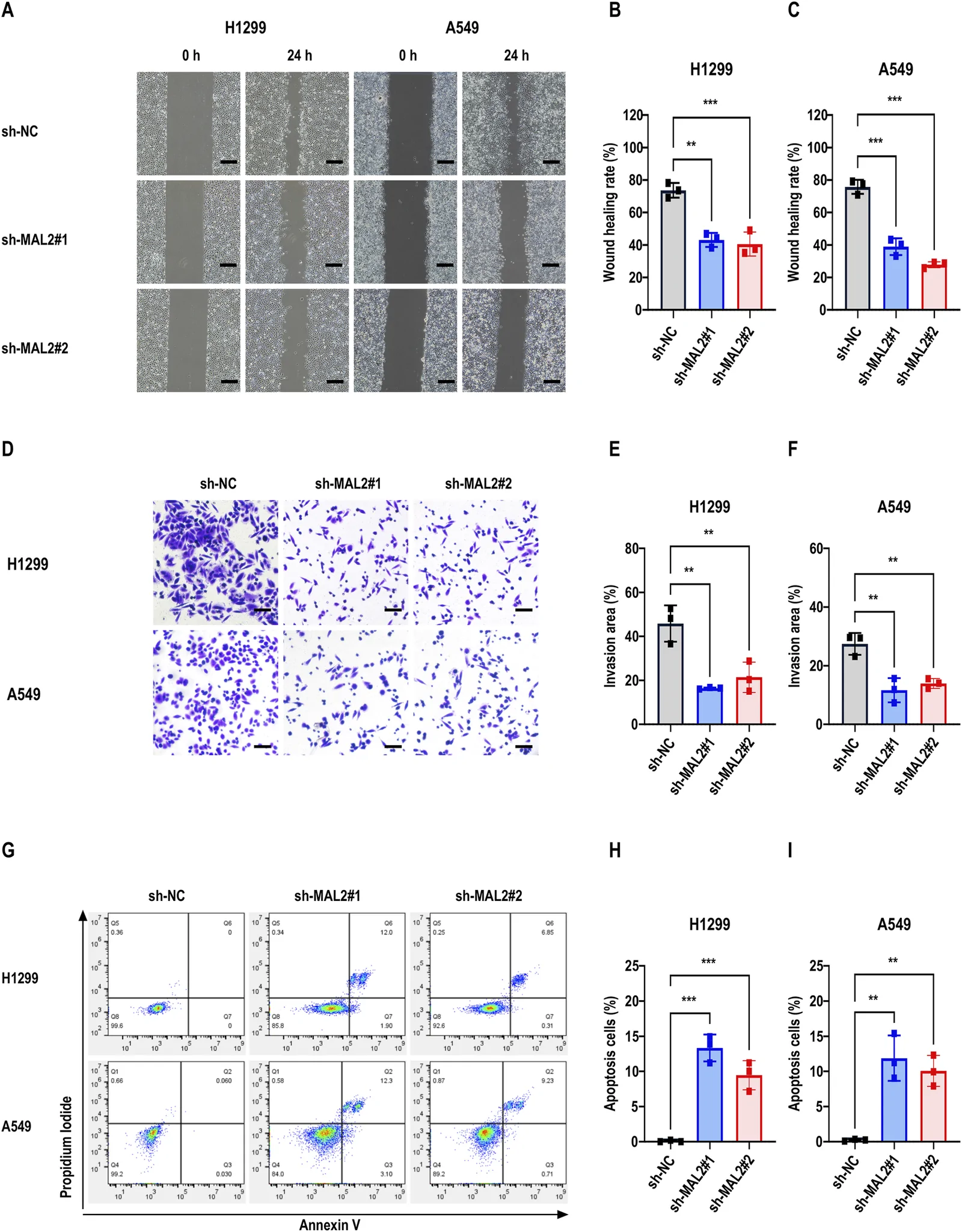
MAL2 depletion inhibits migration and invasion while promoting apoptosis. **(A)** Representative wound healing images of H1299 and A549 cells at 0 h and 24 h. **(B,C)** Quantification of wound healing rates. **(D)** Representative Matrigel-coated Transwell invasion images; columns from left to right are sh-NC, sh-MAL2#1, and sh-MAL2#2. **(E,F)** Quantification of invasion area. **(G)** Representative Annexin V/PI flow cytometry plots. **(H,I)** Quantification of apoptotic cells in H1299 and A549 cells. Data are shown as mean ± SD from three independent experiments (n = 3). Group comparisons used one-way ANOVA with Bonferroni–Šídák adjustment. **P < 0.01, ***P < 0.001. Scale bars: 100 μm in A and 20 μm in **(D)**.

### 3.14 MAL2 knockdown altered HLA-ABC expression and cytokine readouts

Because the computational framework linked MAL2 to immune-resistance states, we next tested whether MAL2 knockdown altered immune-facing tumor-cell features. qPCR analysis showed that MAL2 silencing increased the expression of B2M, CXCL10, and CCL5, while reducing TGFB1 expression in both H1299 and A549 cells (Figures 14A,B). Flow cytometry demonstrated a higher percentage of HLA-ABC-positive cells after MAL2 knockdown (Figures 14C–E), and immunofluorescence staining confirmed stronger HLA-ABC signal intensity in knockdown cells (Figures 14F–H). ELISA analysis of culture supernatants showed that MAL2 depletion reduced relative IL-6 and TGF-β supernatant signals in both cell lines (Figures 14I,J). Together, these assays showed that MAL2 depletion was accompanied by higher HLA-ABC expression and lower relative cytokine signals.

**FIGURE 14:**
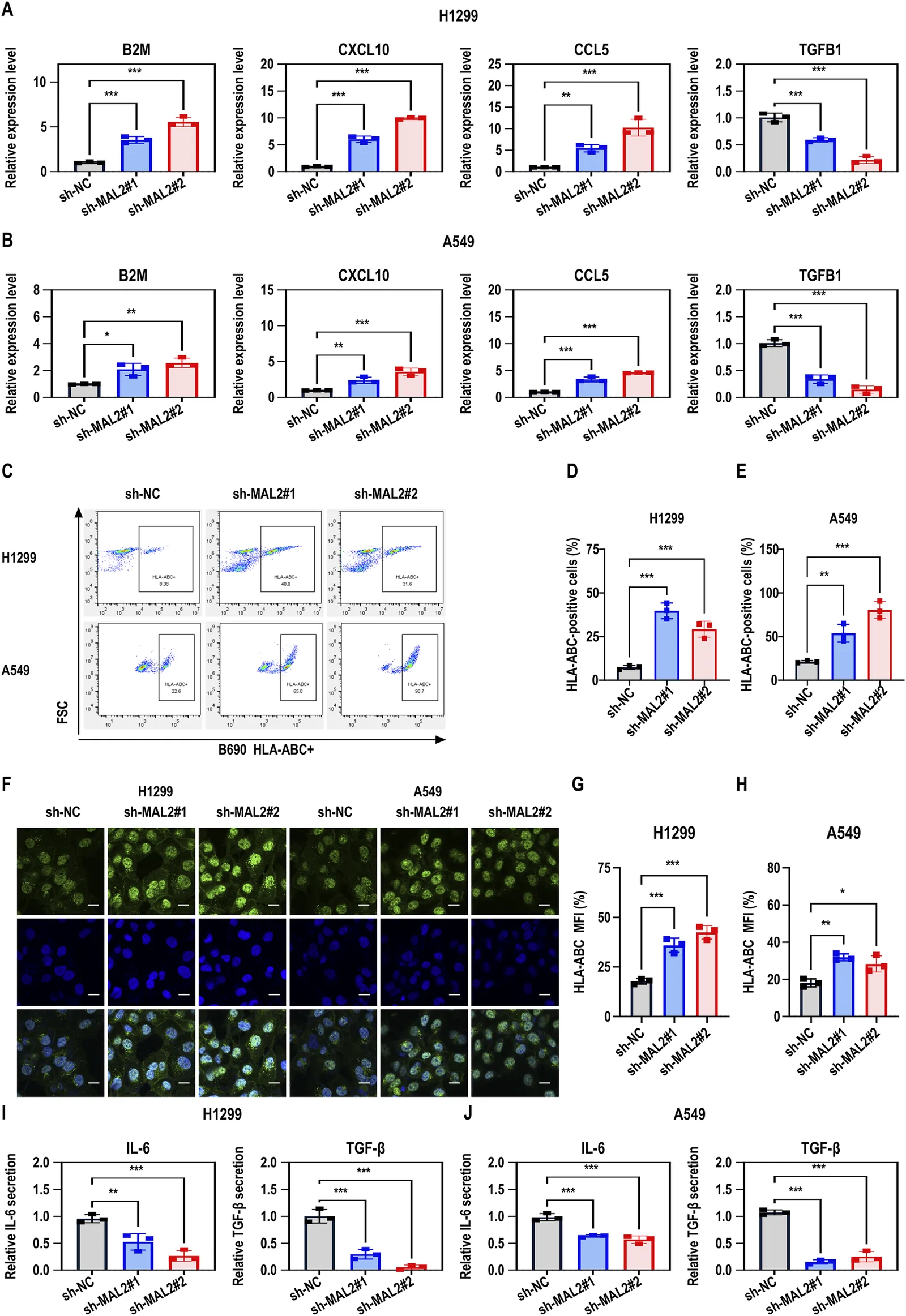
MAL2 knockdown alters HLA-ABC expression and relative cytokine signals. **(A,B)** qPCR analysis of B2M, CXCL10, CCL5, and TGFB1 in H1299 and A549 cells. **(C)** Representative HLA-ABC flow cytometry plots. **(D,E)** HLA-ABC-positive cell fractions. **(F)** HLA-ABC immunofluorescence with DAPI counterstaining; rows show HLA-ABC (green), DAPI (blue), and merged images. Scale bars, 20 μm. **(G,H)** Quantification of HLA-ABC fluorescence intensity in H1299 and A549 cells, respectively. **(I,J)** Relative IL-6 and TGF-β supernatant signals measured by ELISA. Graphs display measurements from three independent experiments (n = 3), with mean ± SD. MFI, mean fluorescence intensity. Endpoint comparisons used one-way ANOVA with Bonferroni–Šídák adjustment. *P < 0.05, **P < 0.01, ***P < 0.001.

### 3.15 MAL2 knockdown altered apoptosis and activation-associated readouts in PBMC co-cultures

In co-culture experiments using sh-MAL2#1, Annexin V/PI profiles and quantitative summaries were obtained for both H1299 and A549 cells (Figures 15A–C). In H1299 cultures, mean Annexin V positivity was 17.14% after knockdown alone, 21.86% with sh-NC plus PBMCs, and 35.06% with knockdown plus PBMCs (Figures 15A,B). In A549 cultures, the mean Annexin V-positive fraction was 0.023% for sh-NC, 10.75% for knockdown alone, 15.82% for sh-NC plus PBMCs, and 31.65% for knockdown plus PBMCs (Figures 15A,C). CD69-positive gated events were more frequent in knockdown co-cultures in both cell-line systems (Figures 15D–F). Relative IFN-γ and TNF-α supernatant signals were also higher in the knockdown-plus-PBMC groups (Figures 15G,H). The co-culture readouts therefore linked MAL2 depletion to increased apoptosis-associated positivity, CD69 expression, and relative inflammatory cytokine signals.

**FIGURE 15:**
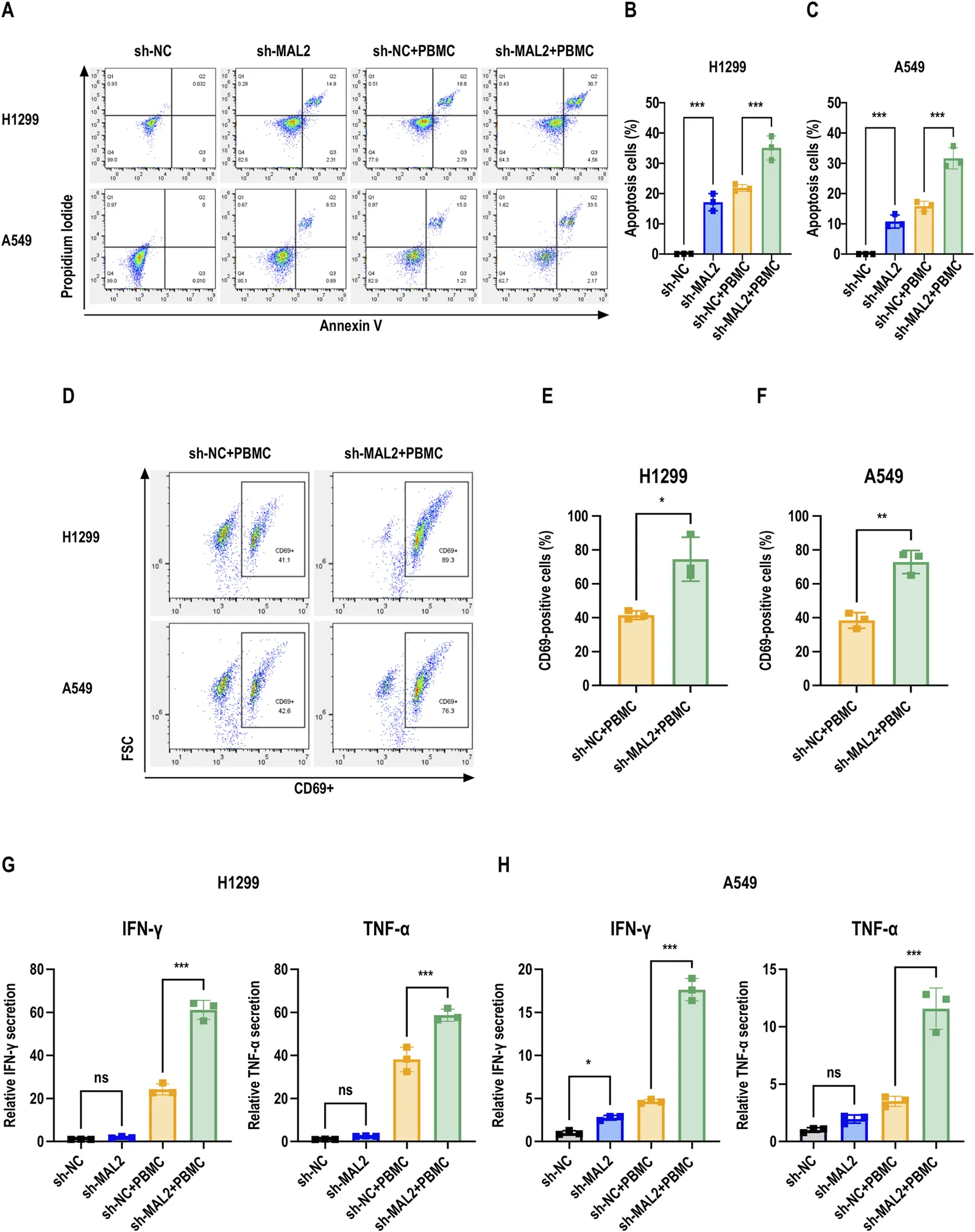
Apoptosis- and activation-associated readouts in PBMC co-cultures. **(A)** Representative Annexin V/PI plots for sh-NC, sh-MAL2, sh-NC plus PBMCs, and sh-MAL2 plus PBMCs in the H1299 and A549 systems. **(B,C)** Corresponding quantitative summaries for H1299 and A549. **(D)** Representative CD69-positive gates in co-culture suspension-cell preparations. **(E,F)** CD69-positive event fractions in the H1299 and A549 systems. **(G,H)** Relative IFN-γ and TNF-α supernatant signals in H1299 and A549 cultures. Here, sh-MAL2 denotes sh-MAL2#1 in both cell lines. Graphs display measurements from three independent experiments (n = 3), with mean ± SD. Apoptosis and ELISA comparisons used one-way ANOVA with Bonferroni–Šídák adjustment; CD69 comparisons used two-sided unpaired Welch’s t tests. ns, P ≥ 0.05; *P < 0.05, **P < 0.01, ***P < 0.001.

## 4 Discussion

Lung cancer remains a major cause of cancer mortality, and immune checkpoint blockade has become a central part of treatment for advanced and resectable NSCLC. Yet durable benefit is still limited to a subset of patients. In this study, we modeled immunotherapy response as a balance between immune activation and tumor-intrinsic resistance and converted this biology into a data-driven biomarker framework. The score was associated with response and PFS in the evaluated cohorts, although its performance was not consistently superior to the immune axis and was weaker in the newly evaluated outcome cohort. This framework nominated MAL2 as a tumor-cell candidate, and *in vitro* depletion altered cell growth, motility-related readouts, and immune-related phenotypes.

The results fit with a broader view of checkpoint response as a composite biological state rather than a property captured by one marker. Reviews of checkpoint biomarkers emphasize that PD-L1, TMB, interferon response, antigen presentation, and immune-cell infiltration each explain only part of the clinical variance (Havel et al., 2019). Resistance can arise before therapy, during adaptive immune pressure, or after initial response, and these phases involve both immune-contexture and cancer-cell programs (Sharma et al., 2017). Our dual-axis score was designed around this principle. It did not treat cytotoxic inflammation as sufficient by itself; instead, it scored immune activity against proliferative and epithelial tumor-intrinsic resistance. This is consistent with the cancer-immunity cycle model, in which antigen release, presentation, priming, trafficking, infiltration, recognition, and killing all have to remain functionally connected for immunotherapy to work (Chen and Mellman, 2013).

The favorable pole of our model was characterized by cytotoxic T/NK activity, antigen/APC signaling, and checkpoint-inflamed programs. These features are biologically plausible. Local immune cytolytic activity has been linked to the expression of granzyme and perforin genes across cancers (Rooney et al., 2015), and pan-cancer immune landscapes show that inflammatory and lymphocyte-rich tumors often occupy distinct clinical and molecular states (Thorsson et al., 2018). At the same time, inflamed tumors are not uniformly responsive. T-cell dysfunction, exclusion, and interferon-related feedback programs can limit the effectiveness of checkpoint blockade even in tumors with substantial immune infiltration (Jiang et al., 2018). The small changes seen when the checkpoint coefficient was varied suggest that it contributed relatively little incremental discrimination within these datasets. In clinical samples, an inflamed signal can represent productive immunity, exhausted immunity, or both, depending on whether antigen presentation and tumor-cell vulnerability are preserved (Chi et al., 2025).

The unfavorable gene list showed weaker and more restricted enrichment than the immune list, with DNA-replication terms providing the clearest evidence. Epithelial localization of selected resistance-module genes provided a separate cellular context for this axis. These signals are less often emphasized than immune infiltration, but they are increasingly recognized as important determinants of immune escape. Cancer-cell-intrinsic programs can shape immune exclusion and checkpoint resistance by altering cytokine gradients, antigen-presentation machinery, tissue architecture, and tumor-cell fitness (Jerby-Arnon et al., 2018). Single-cell studies have also shown that treatment response may involve expansion or replacement of particular T-cell clonotypes rather than a simple increase in total T cells (Yost et al., 2019). From this perspective, tumor-cell state matters because it determines whether immune pressure can be converted into effective killing. Our single-cell analyses localized the relevant expression sources: immune-benefit genes localized largely to lymphoid or myeloid compartments, whereas MAL2 and related resistance candidates localized to marker-defined epithelial compartments.

The integration of bulk and single-cell data was therefore central to the logic of target selection. Bulk transcriptomes are powerful for cohort-level modeling, but they can obscure cellular origin. Single-cell analysis helps distinguish whether a gene is part of an immune response, a stromal program, or a tumor-cell state (Ren et al., 2023). Similar principles have been used to dissect immunotherapy-associated cell populations in melanoma and breast cancer, where specific immune and tumor compartments rather than bulk immune abundance alone were linked to response (Gide et al., 2019; Zhang et al., 2021). In lung cancer, the tumor microenvironment contains extensive stromal, myeloid, lymphoid, and epithelial heterogeneity (Lambrechts et al., 2018). By asking whether candidate genes were detectable and epithelial-enriched, we avoided selecting immune-derived markers that would be difficult to validate in tumor-cell knockdown experiments. Here, however, strong epithelial localization did not imply a within-epithelium response effect: patient-level pseudobulk MAL2 expression was not associated with pathological response. The close relation between overall MAL2 abundance and epithelial fraction instead highlights a composition-related explanation for part of the bulk signal.

MAL2 is biologically credible as a tumor-cell candidate because it is linked to polarized membrane trafficking. Early cell-biology studies identified MAL2 as a raft-associated protein required for basolateral-to-apical transcytosis in polarized epithelial cells (de Marco et al., 2002; de Marco et al., 2006), and MAL2 expression has been described in human epithelia with transcytotic activity (Marazuela et al., 2004). This trafficking function provides a mechanistic context: a trafficking-related epithelial protein could plausibly influence membrane composition, antigen-presentation-related machinery, secretory behavior, or cell-cell interactions. A lung cancer study has reported that MAL2 can facilitate proliferation through mTOR-associated ribosome biogenesis (Lian et al., 2022), which is consistent with our finding that MAL2 knockdown suppressed growth and colony formation in A549 and H1299 cells.

The experiments also linked MAL2 depletion to changes in HLA-ABC expression and cytokine readouts. MAL2 knockdown increased B2M and HLA-ABC expression and decreased TGFB1 expression and relative IL-6 and TGF-β supernatant signals. Loss or impairment of HLA class I antigen processing and presentation is a recognized mechanism of acquired resistance to checkpoint inhibitors in lung cancer (Gettinger et al., 2017), and patient HLA class I genotype can influence response to checkpoint blockade (Chowell et al., 2018). Our HLA-ABC findings are consistent with work in breast cancer showing that MAL2 can drive immune evasion by suppressing tumor antigen presentation (Fang et al., 2021). They extend this question to lung cancer cells, where changes in surface HLA-ABC accompanied reduced growth and increased apoptosis. In PBMC-containing cultures, higher Annexin V and CD69 positivity and increased relative IFN-γ and TNF-α signals indicate changes in the combined cellular response.

The cytokine findings also connect MAL2 to a broader resistance environment. TGF-β signaling is a well-established mediator of immune exclusion and resistance to PD-L1 blockade in several tumor models and clinical settings (Mariathasan et al., 2018; Tauriello et al., 2018). In preclinical models, IL-6 blockade improved tumor control and reduced immune-mediated toxicity during checkpoint inhibition (Hailemichael et al., 2022). In our data, MAL2 knockdown reduced the relative IL-6 and TGF-β supernatant signals while increasing chemokine and antigen-presentation-related signals. These concurrent changes link MAL2 depletion to both cell fitness and the extracellular signaling environment. Distinguishing direct effects on membrane trafficking or secretion from secondary changes in cell state will be important for understanding this relationship. Without normalization to viable cell number, reduced cytokine signal could partly follow the loss of viable cells rather than reduced secretion per cell.

Several limitations affect interpretation. The cohorts were small and retrospective, with different treatments and endpoints. Module membership was discovery-informed, so refitting preprocessing and model tuning within training folds did not remove feature-selection bias. Conditional validation used a 50-gene common subset rather than the complete 54-gene list. GSE190266 had incomplete feature coverage and had contributed unlabeled expression summaries, limiting assessment of the full score in an independent cohort. Single-cell response comparisons involved four favorable and eight unfavorable post-treatment patients, and marker-defined epithelial cells were not classified as malignant by copy-number analysis. MAL2 prioritization was weight-dependent, with weak standalone clinical and within-epithelial response associations. In the experiments, changes in growth and viability could contribute to wound closure and invasion endpoints. Co-culture apoptosis and CD69 positivity were not resolved to tumor cells and CD3-positive T cells, respectively; increased baseline apoptosis after knockdown also complicates attribution of an additional PBMC effect. These assays therefore do not establish tumor-specific killing or antigen-specific recognition. Rescue experiments and antigen-specific assays would be needed to define the mechanism, while independent clinical cohorts are required to assess biomarker performance (Su et al., 2025).

## 5 Conclusion

The immune-resistance framework connected response-associated transcriptional programs with the epithelial candidate MAL2. MAL2 depletion reduced lung cancer cell growth and altered HLA-ABC, cytokine, and PBMC co-culture readouts. These findings support a role for MAL2 in cell fitness and immune-related phenotypes and provide a basis for further mechanistic investigation.

## Data Availability

The original contributions presented in the study are included in the article/supplementary material, further inquiries can be directed to the corresponding author.
